# Phosphorothioate-DNA bacterial diet reduces the ROS levels in *C. elegans* while improving locomotion and longevity

**DOI:** 10.1038/s42003-021-02863-y

**Published:** 2021-11-25

**Authors:** Qiang Huang, Ruohan Li, Tao Yi, Fengsong Cong, Dayong Wang, Zixin Deng, Yi-Lei Zhao

**Affiliations:** 1 State Key Laboratory of Microbial Metabolism, Joint International Research Laboratory of Metabolic & Developmental Sciences, Department of Bioinformatics and Biostatistics, School of Life Sciences and Biotechnology, Shanghai Jiao Tong University, Shanghai, 200240 China; 2 Department of Chemistry, Fudan University, 2005 Songhu Road, Shanghai, 200438 China; 3 College of Chemistry, Chemical Engineering and Biotechnology, Donghua University, Shanghai, 201620 China; 4 National Experimental Teaching Center, School of Life Sciences and Biotechnology, Shanghai Jiao Tong University, Shanghai, 200240 P.R. China; 5 Key Laboratory of Environmental Medicine Engineering in Ministry of Education, Medical School, Southeast University, Nanjing, 210009 China

**Keywords:** Ageing, Bacterial host response

## Abstract

DNA phosphorothioation (PT) is widely distributed in the human gut microbiome. In this work, PT-diet effect on nematodes was studied with PT-bioengineering bacteria. We found that the ROS level decreased by about 20–50% and the age-related lipofuscin accumulation was reduced by 15–25%. Moreover, the PT-feeding worms were more active at all life periods, and more resistant to acute stressors. Intriguingly, their lifespans were prolonged by ~21.7%. Comparative RNA-seq analysis indicated that many gene expressions were dramatically regulated by PT-diet, such as cysteine-rich protein (*scl*-11/12/13), sulfur-related enzyme (*cpr*-2), longevity gene (*jnk*-1) and stress response (*sod*-3/5, *gps*-5/6, *gst*-18/20, *hsp*-12.6). Both the Gene Ontology (GO) and Kyoto Encyclopedia of Genes and Genomes (KEGG) enrichment analysis suggested that neuroactivity pathways were upregulated, while phosphoryl transfer and DNA-repair pathways were down-regulated in good-appetite young worms. The findings pave the way for pro-longevity of multicellular organisms by PT-bacterial interference.

## Abstract (web summary)

Qiang Huang et al. fed *C. elegans* with *E. coli* containing phosphorothioate (PT) DNA or a control strain and evaluated the impact on animal physiology. They observed that worms fed PT( + ) diets exhibited low reactive oxygen species, more active movement, and a longer lifespan compared to controls, suggesting that PT-DNA may have a positive effect on animal health.

## Introduction

Phosphorothioate DNA (PT-DNA), in which a non-bridging oxygen in the phosphate link is replaced by a sulfur atom, is widely distributed among bacteria and archaea, according to the *dnd*A-E functional gene cluster survey^1^. Similar to epigenetic methylation, the physiological DNA phosphorothioation is of specificity in *R*_*p*_*-*chiral modification^2,3^ and G_PS_AAC/G_PS_TTC, G_PS_GCC, and C_PS_CA- consensus sequences (usually 12–14% frequency of these sequence motifs)^4–6^. At present, two biological functions of PT-DNA have been recognized by means of microbial studies: restriction-modification (R-M), to resist the invasive non-PT-modified genetic material of bacteriophages^7–9^; and on-site DNA protection, to counteract ROS assault^10–12^. The R-M system is constrained to the bacterial compartment in the case of intracellular gene-cluster mechanics (either *dnd*F-H or *pbe*A-D)^8,13,14^. However, the anti-ROS “one-atom S/O chemistry” property can be feasibly inherited when other organisms ingest PT-containing bacteria^11,12,15^.

Typically, the S/O anti-ROS mechanism suggests that ROS molecules could be transformed to protective thiyl species whenever phosphorothioate is oxidized to normal phosphate^12^. Besides the straightforward redox function, a combinative analysis of epigenome, transcriptome, and metabolome has indicated that PT modification also contributes to the cellular redox state and stress resistance in bacteria^1^. Intriguingly, Wu et al. discovered physiological phosphorothioate RNA in both prokaryotes and eukaryotes, *e.g*., HeLa cells, with highly sensitive liquid chromatography coupled with tandem mass spectrometry^16^. Recently, a human microbiome study showed that over 2000 PT-gene-containing bacterial strains, including the genera *Pseudomonas*, *Clostridioides*, and *Escherichia*, were distributed in the gastrointestinal tract and six PT-dinucleotides (C_PS_G, C_PS_T, A_PS_G, T_PS_G, G_PS_C, and A_PS_T) were detected in human fecal DNA^17^. These discoveries demonstrate the existence of DNA/RNA phosphorothioation in both prokaryotes and eukaryotes, and it is worthwhile to understand the possible long-term diet effects of PT-containing bacteria.

Currently, many anti-ROS phenotypes and molecular mechanisms have been investigated for PT-containing bacteria. Wang et al. have reported that catalase and organic hydroperoxide resistance gene expressions were not up-regulated in the PT-containing wild type *Streptomyces lividans*^18^, though the PT-containing bacteria exhibited 2 to 10-fold higher survival under peroxide oxidative stress and its ROS scavenging was much faster than the *Dnd-* mutant. Furthermore, PT modification plays a dual role in protecting and damaging genome—the PT site sacrifices itself to generate a sulfur-derived ROS scavenger, but simultaneously increases the chance of DNA fragmentation at the PT site during the antioxidative process^12,19^. The fragmentation-after-acting characteristic promotes PT-containing DNA fragments release from dead bacteria to environment under oxidative stress, and increases its intake by surrounding bacteria or as nutrients ingested by host organisms. Beyond anti-oxidative stress, PT-containing bacteria also exhibited resistance to heavy metals, and to ultraviolet and X-ray radiation^11,12,18,20^. Particularly, previous studies have shown that phosphorothioate plays a mild protective role, without pro-oxidative side-effects^11^. It is worth knowing whether the host can benefit from the long-term diet intake of phosphorothioate as well.

In this study, we fed the wild-type *C. elegans* strain (Bristol N2) either PT-bioengineered *E. coli* OP50 or a control. Then the ROS level and lipofuscin accumulation, body and brood sizes, locomotion, and lifespan were compared between the two groups of nematodes, and RNA-seq analysis was carried out at the most discriminating time points (four-day and twelve-day old, D4 and D12) to discern any change in gene-expression. It was observed that the ROS level substantially decreased in *C. elegans* fed PT-containing bacteria, and the worms became healthier in terms activity and longevity. Furthermore, the PT-fed worms exhibited tolerance to environmental stresses caused by paraquat, heat, and Cr^6+^, but not for ultraviolet radiation. Our results suggest that *C. elegans* ingest phosphorothioate from PT-bacterial food. Unlike the bacterial transcriptome^18^, the *C. elegans* transcriptome exhibited statistically meaningful regulation of the expression of many genes, including but not limited, to neuroactivity, DNA repair, phosphoryl transfer, defense-response, and sulfur-related metabolism. It is likely that the bacteria-sourced phosphorothioates take part in multiple pathway-network regulations and thereby influence the observed phenotypes, such as locomotion and aging.

## Results

### PT-containing bacteria food reduces the ROS level in *C. elegans*

**Fig. 1:**
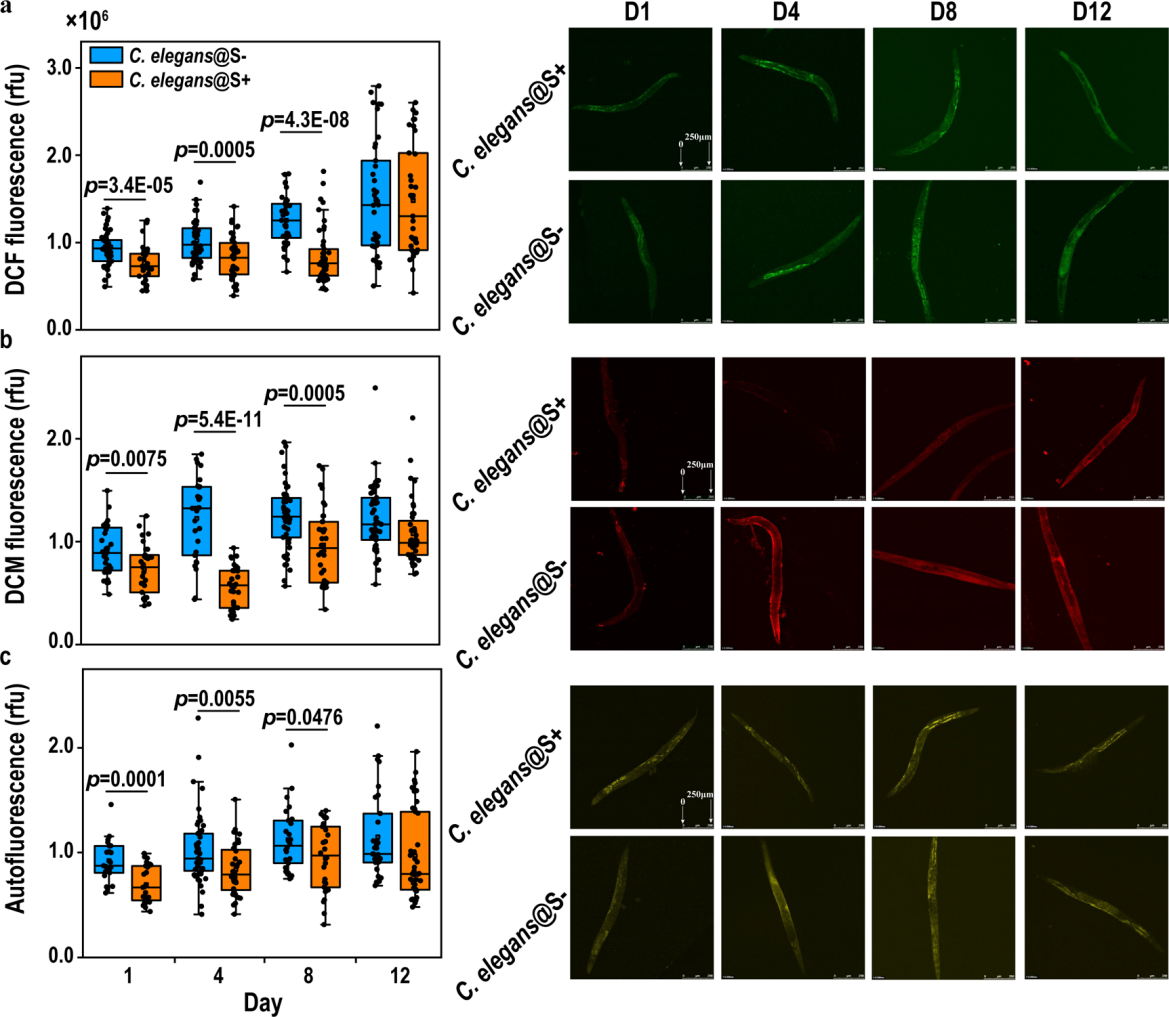
The ROS level and lipofuscin accumulation of *Caenorhabditis elegans* fed Dnd + (S + ) and Dnd- (S-) OP50. **a** ROS level measured by the fluorescent probe H_2_DCF-DA (excitation, 488 nm; emission 550-600 nm, *n*_S+_ > 35, *n*_S-_ > 36); **b** ROS level measured by the fluorescent probe DCM (excitation, 478 nm; emission 550-600 nm, *n*_S+_ > 28, *n*_S-_ > 27); and **c**) Autofluorescence of lipofuscin (excitation, 478 nm; emission 630–700 nm, *n*_S+_ > 24, *n*_S-_ > 26). *C. elegans* samples were collected on Day 1, 4, 8, and 12, after L4 larva-to-adult molts at 22 ^o^C, the images with fluorescence close to average reads were selected as examples in Fig. 1. Central lines represent the mean values, and error bars note the standard deviation (rfu, relative fluorescence units. Statistical significance was calculated with two-tailed t-test and *p* values are indicated. see the details in Supplementary Figs. 1–3).

The most remarkable and straightforward long-term diet effect was on the ROS level in *C. elegans*. Figure 1 compares the ROS levels and lipofuscin accumulation of *C. elegans* fed with *E. coli* OP50 harboring a pBluescript-based plasmid (pJTU1238) (S + , Dnd phenotype positive), which has the *dnd* gene cluster from *Salmonella enterica*, and normal *E. coli* OP50 (S-, Dnd phenotype deficient, negative control). Plasmid DNA from the two *E. coli* OP50 strains were tested by using iodine-induced PT-DNA cleavage to confirm PT-modification in the bacteria^12^. To examine the long-term effect of exogenous phosphorothioate on *C. elegans*, the wide type Bristol N2 nematodes were fed *E. coli* OP50 S + (Dnd + ) and normal OP50 (S-, Dnd-).

As shown in Fig. 1a and Supplementary Table 1, the H_2_DCFDA-related ROS level decreased by 18.8, 19.1, and 32.7% at day 1, 4, and 8, respectively, while it slightly decreasing by 0.9% at day 12. Likewise, the DCM-related level respectively decreased by 19.9, 54.0, and 21.5% at day 1, 4, and 8, but no significant change at day 12. The *p*-values for the lower ROS levels in the PT-fed nematodes were less than 0.001 in the D4 and D8 samples by the DCM measurement, and less than 0.01 by the DCF measurement. No statistically significant ROS drop was observed for the D1 and D12 samples.

The 2′, 7′-dichlorodihydrofluorescein diacetate (H_2_DCFDA) fluorescent probe passively diffused into cells and reacts with most ROS species, including hydrogen peroxide, the hydroxyl radical, and peroxynitrite after cleavage by in vivo esterase^21,22^. Upon oxidation by an ROS species, the nonfluorescent H_2_DCFDA was converted to 2′, 7′-dichlorofluorescein (DCF), and thereby, the DCF fluorescent intensity could be a linear response to the ROS molecules in *C. elegans*^23–25^. Similarly, the nonfluorescent 2-(2-(4-(4,4,5,5-tetramethyl-1,3,2-dioxaborolan-2-yl)styryl)-4H-chromen-4-ylidene)malononitrile (i.e., DCM boronate) was converted to fluorescent dicyanomethylene-4H-pyran fluorophore; but the DCM fluorogenic probe was more sensitive to hydrogen peroxide and peroxynitrite^26,27^. Interestingly, ROS quantification appeared more dramatic when measured by DCM for *C. elegans* fed with PT-containing OP50, even though no NOS gene (to biosynthesize nitric oxide, a common peroxynitrite precursor combining with superoxide) existed in N2 *C. elegans* or *E. coli* OP50^28^.

ROS suppression by PT-containing bacterial diet was in a good agreement with previous studies of phosphorothioate antioxidant properties^10–12^. Typically, the S/O anti-oxidant mechanism is also ROS species-related—the PT-genome is highly sensitive to some ROS species like hypochlorous acid^19^, whereas phosphorothioation is more protectable in the presence of peroxynitrite and hydrogen peroxide^11,12^. One rationale is that competitive P-O and P-S cleavages occur upon ROS attack. If the double-stranded P-O cleavage occurs on the PT site, then the bacterial genome would be fragmented and apoptosis. On the other hand, if only the P-S bond is broken by a ROS species, then the resulting thiyl derivatives would scavenge the surrounding ROS species. For an extreme example, iodine (I_2_) can effectively crack the bridging P-O bonds at the PT-modified site of PT-DNA and is widely used to detect the PT-DNA phenotype^5^. Hydrogen peroxide and peroxynitrite prefer the PS/PO conversion via P-S cleavage at the PT-modified site. Thus, the low ROS level in the PT-fed nematodes indicates that phosphorothioate may act as an anti-ROS species after digestive intake.

Figure 1c shows the age-accumulation of fluorescent material in *C. elegans*, which was usually considered as autofluorescent lipofuscin from the aging process. In wild-type animals, age pigments increase into adulthood, accumulating slowly during the reproductive phase and more rapidly during the post-reproductive period^29–31^. Thus, lipofuscin accumulation indicates a physiologically aged state, rather than simply marking chronological time. The lipofuscin accumulation of the nematodes fed with PT-containing bacteria was much slower than those with normal OP50 diet. Therefore, a delayed rate of aging coincides with the consumption of PT-containing food, according to the lipofuscin biomarker measured by autofluorescence.

### Phenotype changes potentially caused by PT-containing food

**Fig. 2:**
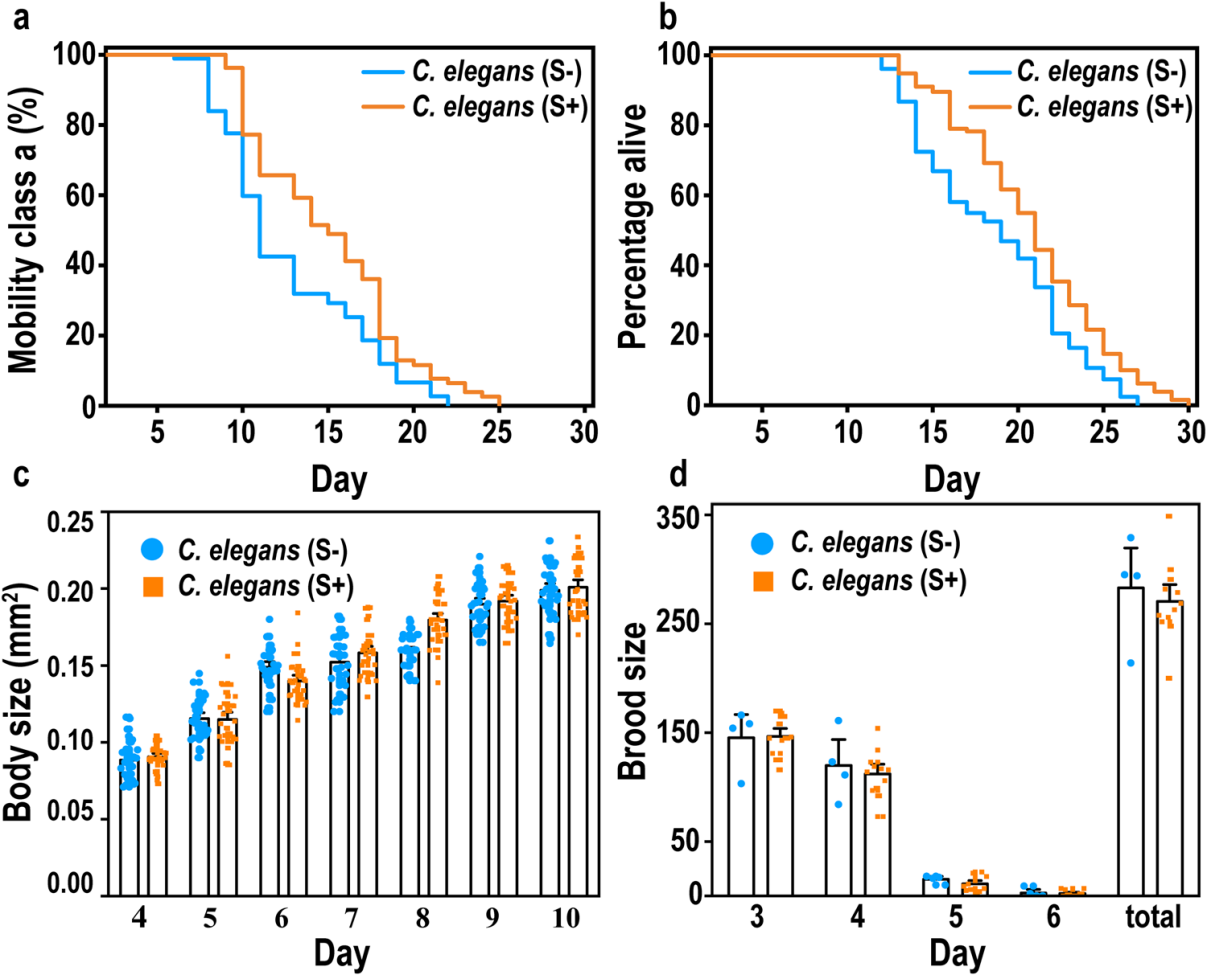
Phenotypic changes observed for *Caenorhabditis elegans* fed PT-containing food after the L4-to-adult molt. **a** The percentage of nematodes with class *a* motility ((S+)-fed *C. elegans* were more active; *p*-value < 0.01, n_S+_ = 78, n_S-_ = 75). **b** Percentage alive in the two groups of worms, observed from the nominal age of 3 days ((S+)-fed *C. elegans* had longer lifespans; *p*-value < 0.01, n_S+_ = 132, n_S-_ = 124). **c** Body sizes of the two groups of nematodes (no significant change; n_S+_ > 14, n_S-_ > 20). **d** Brood sizes of the two groups of nematodes (no significant change; n_S+_ = 8, n_S-_ = 8). *p*-values were calculated with log-rank test.

The locomotion, lifespan, body, and brood sizes of *C. elegans* (Bristol N2 strain) post L4-to-adult molt are illustrated in Fig. 2, with comparison of PT-containing bacterial food and normal OP50 food.

The motility of worms was classified into four ranks: (*a*) robust, coordinated sinusoidal locomotion; (*b*) uncoordinated and/or sluggish movement; (*c*) no forward or backward movement, but head movements or shuddering in response to prodding; and (*d*) dead animals^32,33^. Figure 2a shows the percentage of class *a*—those that exhibited spontaneous movement or vigorous locomotion in response to prodding (also see the percentages of classes b–d in Supplementary Fig. 4). The worms fed with PT-containing bacteria were much more active, particularly during day 10 to 16 (class *a* difference: 19.9 ± 3.0%). All the motility scores of animals fed with PT-containing food were equal to or higher than control at every age tested.

The percentage of nematodes remaining alive in the two groups were recorded under normal culture conditions (*n*_total_ > 250). The lifespans of the animals fed with Dnd + *E. coli* OP50 (S+) were significantly longer than those fed with normal *E. coli* OP50 (Fig. 2b and Supplementary Table 2). The mean lifespan of the two groups of worms were 20.2 ± 1.4 and 16.6 ± 0.8 days respectively (those fed PT-containing bacteria had a 21.7% longer lifetime). To ensure the rigor of the experiment, long-lived (*daf-2, n* *=* *117*) and short-lived (*daf-16, n* *=* *114*) *C. elegans* were cultured under the same conditions with normal *E. coli* OP50 diet. The resulting lifespans were 30 and 13 days on average, respectively (*p*-value < 0.001, see Supplementary Table 2 and Supplementary Fig. 5). Thus, the exogenic effect of food (~3.6 day) on lifespan was much less than the genetic effects (e.g., *daf-2* mutants live longer by 13.7 days, and *daf-16* shorter by 3.4 days under the same experimental condition). Heat-killed bacteria have the similar diet effect on the lifespans of nematodes, confirming the PT-diet effects on their longevity and also suggesting PT-bacteria function as food rather than live gut microbe. (See Supplementary Tables 3–4 and Supplementary Figs. 6 and 7.) Even though both the alive and heat-killed bacteria prolong nematodes’ lifespans more or less, the degree of lifespan change is different: for the long-lived *daf*-2 mutant, the PT-diet enhancement is 5.0 and 4.3% with the alive and heat-killed bacteria, respectively (*p*-value, 0.01–0.02); for the short-lived *daf*-16 mutant, the PT-diet enhancement is 13.6 and 13.0%, respectively. (*p*-value, 0.003–0.03).

As shown in Fig. 2c–d, the body and brood sizes of the two types of nematodes were not significantly different. The biological-engineering diet intervention had little effect on body development.

Taken together, the above phenotypic findings indicate that PT-containing bacterial food was coincident with increased motility and longevity of *C. elegans*, but they aren’t having more offspring and their body sizes are the same.

### Stress responses for exogenous stimulus

**Fig. 3:**
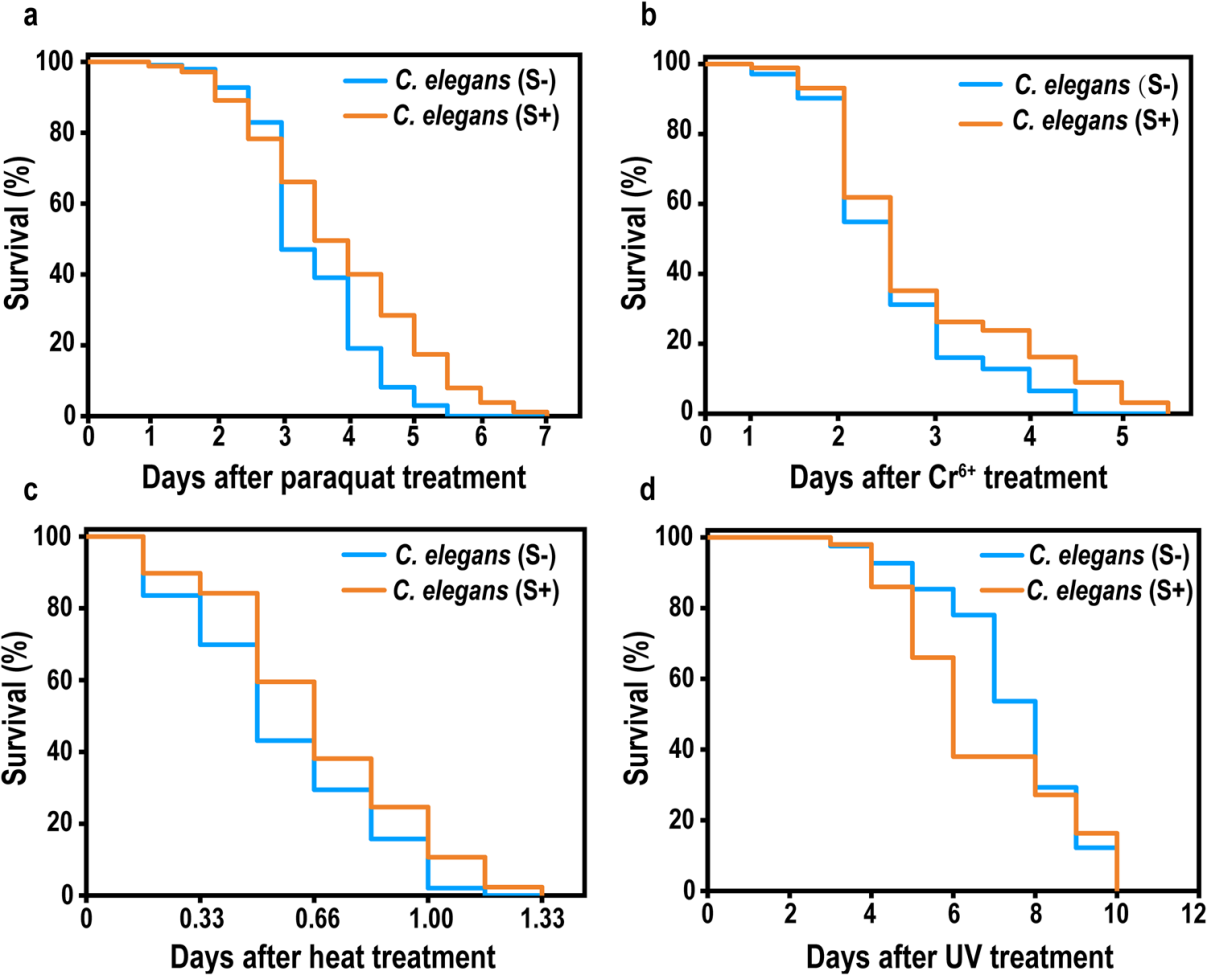
Susceptibility of the two groups of nematodes to acute stresses. Survival percentages after treatment with (**a**) paraquat ((30 mM n_S−_ = 138, n_S+_ = 146, *p*-value < 0.05), (**b**) Cr^6+^ ((10 mM n_S−_ = 111, n_S+_ = 109, *p*-value < 0.05), (**c**) heat shock (35 °C for 6 h, n_S-_ = 127, n_S+_ = 136, *p*-value < 0.01), and (**d**) ultraviolet irradiation (n_S-_ = 41, n_S+_ = 38, *p*-value < 0.001). Animals were exposed to the stresses on day 3 after L4-to-adult molts, and *p*-values were calculated with the log-rank test.

A variety of studies have demonstrated that various kinds of stressors exert deleterious effects by increasing ROS production^20,34–37^. It is worth testing whether the diet effect is also protective in cases of acute oxidative injury. As shown in Fig. 3a–d, paraquat, K_2_Cr_2_O_7_, heat shock, and ultraviolet radiation were used to evaluate responsiveness to acute oxidative stress in *C. elegans*. The survival rates were recorded hours after the stresses imposed upon the two groups of nematodes.

The mean lifespans of *C. elegans* S+and *C. elegans* S− were 93.3 ± 31.8 and 83.2 ± 20.0 h after paraquat poisoning, and 71.5 ± 31.6 and 60.9 ± 21.8 h after Cr^6+^ poisoning, respectively (see Supplementary Table 5). The PT-containing diet may have helped in the cases of paraquat and Cr^6+^, (survival increased by 12.1 and 17.4% on average, respectively. *p*-value < 0.05), and more so against heat shock (17.4 %, *p*-value < 0.01). However, there may be adverse side-effects associated with exposure to ultraviolet radiation while on the long-term S+diet (survivals decrease by 15.6%, *p*-value < 0.001).

Survival percentages associated with long-term consumption of PT-containing bacterial food were likely related to the antioxidant effects of phosphorothioate compounds (see Supplementary Table 6 and Supplementary Fig. 8). Under the same chemical stresses caused by paraquat, if in the presence of phosphorothioate diethyl ester (DEPT), the survival percentages increased significantly by 23.7 as well—but DEPT is noneffective for Cr^6+^-induced stress. Interestingly, PT-containing plasmids were photo-sensitive and underwent photolysis whereas Dnd + bacteria appeared to have stronger resistance to ultraviolet radiation compared to Dnd- bacteria (see Supplementary Fig. 9)—the self-sacrifice of PT-DNA protected the neighboring organisms from the UV damage. This duality implied that PT-containing diet can help *C. elegans* resist certain ROS species, but perhaps not all of them.

Thus, it is quite reasonable to assume that the low ROS level in PT-fed nematodes stemmed from the long-term intake of phosphorothioate, which also helps to prolong the lifespan and promote their motility. However, the intake of phosphorothioate may lead to more complicated effects in *C. elegans*, at the level of the transcriptome and metabolome.

### Comparative transcriptome analysis of *C. elegans* fed PT-containing food

Since phenotypic investigation revealed the most essential changes in young and middle-aged nematodes fed with PT-containing OP50, RNA sequencing was performed on the 4^th^ and 12^th^ day after adulthood, as representative of the youth and early aging stage, respectively^38^.

**Fig. 4:**
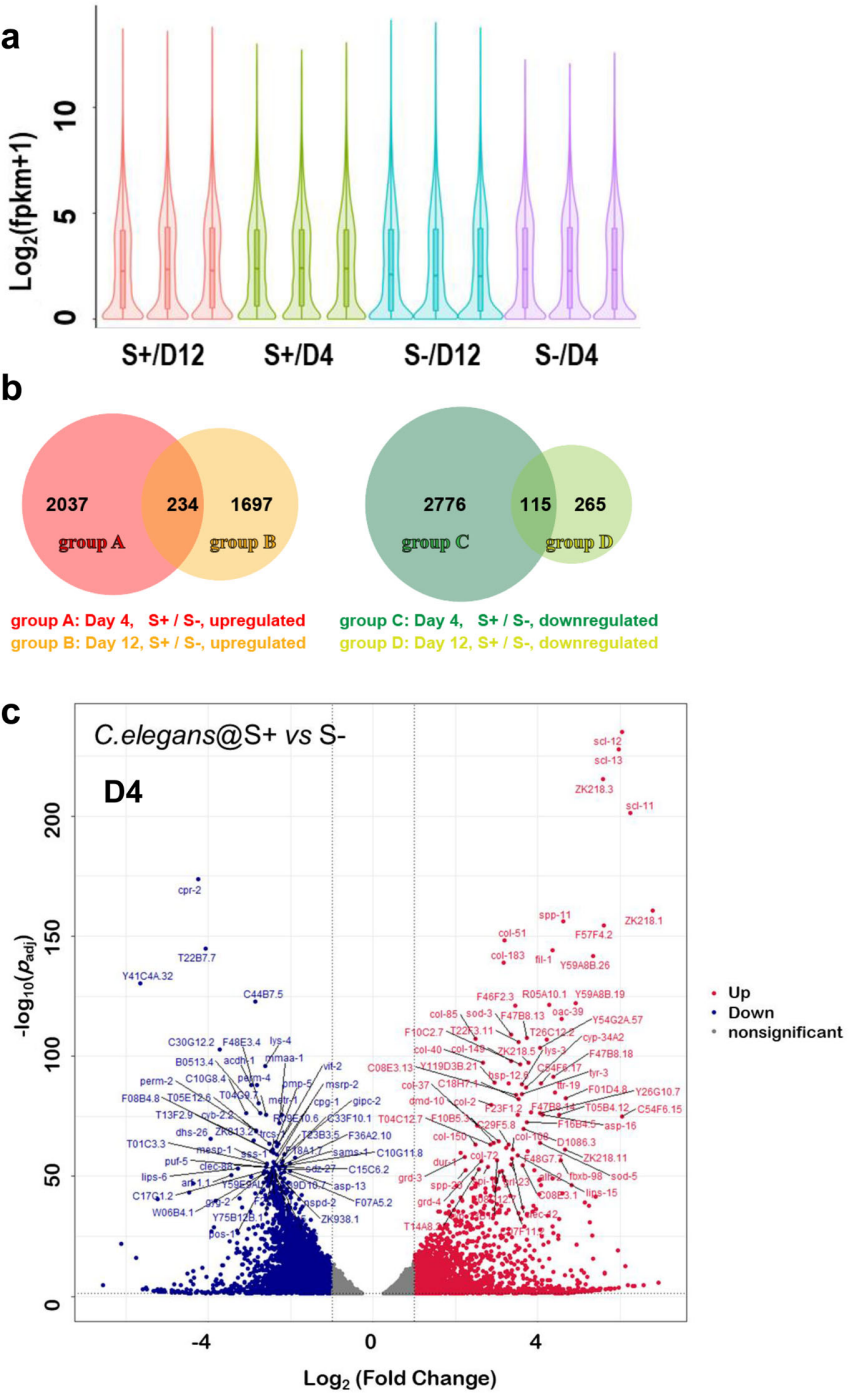
Overview of RNA-seq results. **a** The FPKM distributions in the 12 samples (3 replicates for each group). **b** Venn diagram of differential gene expression between the two groups of *Caenorhabditis elegans* in RNA-seq. **c** Volcano plot of transcriptome differences between *C. elegans* S + and S-, in the D4 samples.

As shown in Fig. 4a, each group of samples gave high-quality FPKM (fragments per kilobase of transcript per million mapped reads) in RNA-seq. The PT-feeding groups presented relatively higher FPKM values in a range of 2^2^–1 to 2^5^–1, in particular for the D4 samples (see Supplementary Fig. 10). In Fig. 4b and Supplementary Table 7, compared to the non-PT-fed nematodes, more genes were transcribed in those that were PT-fed, in both the D4 and D12 samples (see the detailed D4–D12 comparisons in Supplementary Figs. 11–16 and Supplementary Tables 8–14). The number of differentially expressed genes (DEGs) in the D4 samples were more than those in the D12 samples, especially for down-regulated genes. On average, 2271 and 1931 genes were upregulated by nematodes that consumed PT-containing bacterial food in the D4 and D12 samples (|Log_2_FoldChange| > 1 and adjusted *p* value *p*_adj_ < 0.01), respectively. On the other hand, 2891 and 380 genes were down-regulated at the same time. Only 234 of the upregulated genes and 115 of the downregulated genes overlapped between the D4 and D12 samples, indicating the PT-containing food had different effects depending on the age of *C. elegans*^39,40^. It is likely that long-term PT-containing food affects young *C. elegans* more significantly than the middle-aged worms.

In particular, a few outlier genes with extremely small *p*_adj_-values and extremely large fold changes are listed in Table 1.

**Table 1:** The top-ranked up- and down-regulated genes in D4 samples of *Caenorhabditis. elegans*, which were fed PT-containing bacterial food.

Gene	Annotation	F.C.	*p*_adj_	GAAC + GTTC
up-				
*scl*-11	Cysteine-rich secretory protein	76.3	6.0 × 10^−202^	(22 + 21)/4847 bp
*scl*-12	Cysteine-rich secretory protein	65.7	9.3 × 10^−236^	(21 + 14)/4847 bp
*scl*-13	Cysteine-rich secretory protein	62.2	1.7 × 10^−228^	(16 + 20)/4857 bp
ZK218.3	Affected by *lin*-15B, *mex*-3, *dpy*-21	48.2	4.6 × 10^−216^	(25 + 21)/4970 bp
down-				
*cpr*-2	Cysteine-type endopeptidase	1/19.0	1.9 × 10^−174^	(20 + 20)/5175 bp
Y41C4A.32	Affected by *daf*-2, *rrf*-3, *dpy*-10	1/50.3	6.4 × 10^−131^	(13 + 14)/5902 bp
T22B7.7	Acyl-CoA thioesterase	1/16.7	1.7 × 10^−145^	(30 + 20)/6210 bp
C44B7.5	Affected by *daf*-16, *daf*-2, *rrf*-3	1/7.3	1.5 × 10^−123^	(22 + 12)/5218 bp

Annotations were collected from https://wormbase.org. The GAAC/GTTC fragments were searched for using the unspliced transcript plus, upstream and downstream.

Intriguingly, the biological function of top-ranked up- and down-regulated genes pointed to many cysteine-related (i.e., sulfur-related) enzymes and lifespan-related *daf*-2/16 genes; their regulator genes such as *cep*-1, *pal*-1, and *vrk*-1 are systemically down-regulated while *gbb*-1 is up-regulated. (See Supplementary Table 15.) It was very rare to observe genes that were up- or down-regulated by more than 50 times in gene expression, such as *scl*-11/12/13 (cysteine rich protein) and Y41C4A.32 (*daf*-2 related) transcripts. In a recent study of mitochondrial longevity regulated by modulating ER genes, transcript Y41C4A.32 was affected by *isp*-1 and *nfyb*-1^41^ and thereby involved in *daf*-2/insulin-IGF signaling of long-lived mutants. In this work, Y41C4A.32 was down-regulated by ~50 fold in the D4 samples, but slightly up-regulated by 1.3-fold in the D12 samples, reflecting a significant fluctuation during aging. To date, we do not know whether these changes in the transcriptome were directly connected to phosphorothioate as in antisense drugs or not, for lack of detailed information about phosphorothioate nucleotides in the organism.

### Gene expression related to the stress responses and aging

Inspired by the RNA-seq overview analysis^42–46^, we focused on the D4 samples to mine clues about anti-ROS and anti-aging functions.

**Fig. 5:**
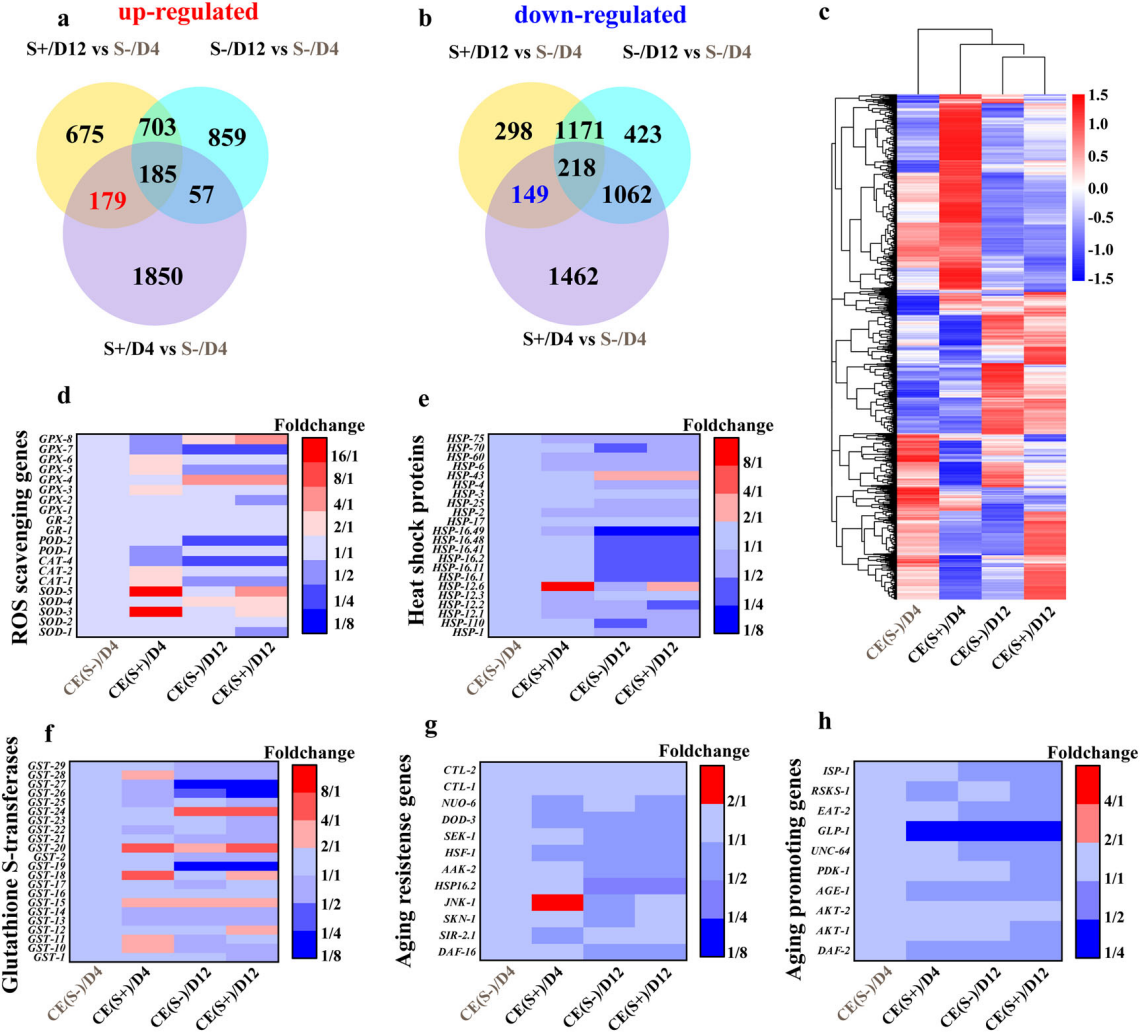
Stress-response and aging-related genes regulated by PT-containing diet. **a**, **b** Venn diagrams of up- and down-regulated genes of the *Caenorhabditis elegans*, using S-/D4 as a reference for the other three groups of samples. **c** Fold-change heatmap of overall gene clustering. **d**–**h** Fold-change heatmaps of the genes for ROS-scavenging, heat-shock protein, glutathione S-transferase, age resistance and promotion for *C. elegans*, with reference to the *C. elegans* S-/D4 samples.

As shown in Fig. 5a–h and Supplementary Table 16, *sod-3*, *sod-5*, *gpx-5*, and *gpx-6* were increased by 10.3, 16.7, 2.3, and 2.3-fold in the *C. elegans* S+/D4 samples compared to the *C. elegans* S-/D4 (control). In addition, the expression of heat shock protein-12.6 (*hsp-12.6*) increased by 9.7 and 5.3-fold in the D4 and D12 samples, respectively. It was known that *sod*, *gpx*, and *hsp* genes were related to antioxidant functions. This suggested that bacterial phosphorothioate not only scavenged ROS in *C. elegans* directly, but also activated these anti-ROS genes in the worms. It should be noted that the anti-ROS gene overexpression is unlikely caused by the reduced ROS level, probably due to the complicated global transcriptomic regulations^47–49^. Interestingly, many expressions of glutathione S-transferases (GST) genes were induced in both the D4 and D12 samples (see Fig. 5f), indicating that the long-term intake of PT-containing food may change sulfur metabolism in the nematodes and induce the expression of GST, which may lead to life extension by increasing antioxidant activity.

Figure 5g–h shows a few aging-related genes with different expressions in the PT-fed and control groups. The stress-response gene *jnk*-1 increased by 2.2-fold and the aging-promoting gene *glp*-1 decreased by 2.9-fold, which might be related to the change in lifespan. Indeed, the aging-related gene analysis indicates the insulin/IGF-1 (IIS) and dietary restriction pathways might involve in the PT-induced longevity effect, because PT-regulated expressions of *mtl*-1, *col*-10, *col*-13, *daf*-12, *jnk*-1, *pha*-4, *ttx*-1, *ser*-3, *daf*-18, *mes*-4, *ins*-7, *sams*-1, and *glp*-1 are anti-aging (see Supplementary Table 17).

Quantitative real-time PCR (qRT-PCR) validated PT-diet regulation on gene expressions discovered by the RNA-seq results. The regulatory patterns of *scl*-11, *scl*-12, *scl*-13, ZK218.3, *cpr*-2, Y41C4A.32, T22B7.7, C44B7.5, *sod*-3, *sod*-5, *gpx*-3, *gpx*-5, *gpx*-6, *cat*-1, *cat*-2, *hsp*-12.6, *gst*-10, *gst*-15, *gst*-18, *gst*-20, *gst*-28, *jnk*-1, *glp*-1 measured by the qRT-PCR were in agreement with the transcriptomic changes measured by the RNA-seq method (correlation coefficient, *R*^2^ = 0.9476) (see Supplementary Figs. 17 and 18 and Table 18).

Thus, PT-containing bacterial food may act as antioxidant directly to oxidative stress and substantially change many gene expressions by the involvement in the nematodes’ transcriptomic network. Interestingly, certain antioxidant enzymes like SODs and GSTs were overexpressed significantly, different from the reduced expression in PT( + ) bacteria under oxidative stress^18^.

### Global gene ontology (GO) enrichment of PT-feeding *C. elegans*

**Fig. 6:**
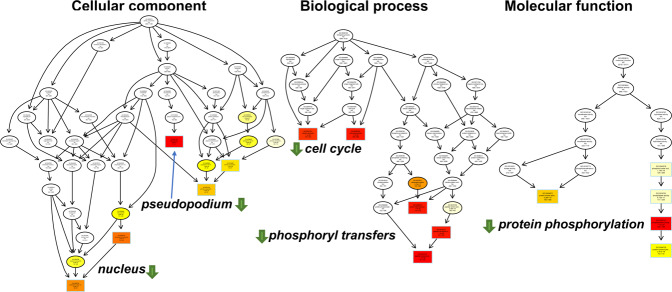
GO enrichment of the differential transcriptomes between the S + and S- samples at Day 4. The GO Directed acyclic graph (DAG) shows the enriched GO terms in the downregulated cellular component, biological process, and molecular function categories. The boxes and circles represent GO terms and are labeled with the GO ID on the first line, the term definition and information on the second line and the *p*-value on the third line. The fourth line is labeled with the number of regulated genes associated with the listed GO ID and the total number of genes. Annotation moves from more general to more specific with progression from the parent nodes. The top-ranked terms with the lowest *p*-values are marked with boxes and yellow/red colors. The more red-saturated a node is, the greater its significance.

Figure 6 illustrates the GO enrichment of the differences between the transcriptomes of *C. elegans* S+/D4 and *C. elegans* S-/D4 samples. Overall, 101, 383, and 123 categories were enriched in ‘cellular component’, ‘biological process’, and ‘molecular function’, while only 20, 43, and 26 categories were enriched in the corresponding D12 samples (Supplementary Data 1–4). This indicates that the long-term PT-containing food had a greater impact on young nematodes, which may have been caused by the nematodes eating more food at a young age. For clarity, the top-rank differential GO terms are listed in Table 2.

**Table 2:** The top-ranking GO terms in the GO enrichment of *Caenorhabditis elegans* fed PT-containing bacterial food, long-term.

GO ID	Description	up	down	p_adj_
Down-regulation				
Cell component				
GO:0031143	pseudopodium	1	36	1.6 × 10^−17^
GO:0000793	Condensed chromosome	0	73	1.1 × 10^−13^
GO:0000794	Condensed nuclear chromosome	0	39	4.2 × 10^−12^
Biological process				
GO:0018107	Peptidyl-threonine phosphorylation	1	80	1.7 × 10^−23^
GO:0018210	Peptidyl-threonine modification	1	80	8.7 × 10^−23^
GO:0006470	Protein dephosphorylation	4	111	1.3 × 10^−25^
Molecular function				
GO:0004721	Phosphoprotein phosphatase activity	4	105	1.1 × 10^−23^
GO:0004672	Protein kinase activity	37	148	3.3 × 10^−19^
GO:0004725	Protein tyrosine phosphatase activity	5	112	2.4 × 10^−18^
Up-regulation				
Cell component				
GO:0005581	collagen trimer	52	22	8.6 × 10^−8^
GO:0044441	ciliary part	32	2	1.1 × 10^−3^
GO:0044463	cell projection part	74	7	1.3 × 10^−3^
Biological process				
GO:0007218	neuropeptide signaling pathway	49	1	2.5 × 10^−4^
GO:0007602	phototransduction	10	0	8.3 × 10^−4^
GO:0009583	detection of light stimulus	10	0	8.3 × 10^−4^
Molecular function				
GO:0042302	structural constituent of cuticle	48	22	9.5 × 10^−7^
GO:0019825	oxygen binding	22	0	1.2 × 10^−4^
GO:0046906	tetrapyrrole binding	49	11	1.6 × 10^−4^

As shown in Table 2, a few genes with phosphoryl transfer-related GO terms (0018107, 0006470, 0004721, and 004725) had statistically significant regulation. It appears likely that phosphorothioate is involved in phosphoryl-transfer processes in the nucleus, e.g., phosphorylation and dephosphorylation of enzymes and kinases. Intriguingly, for some reason, most of the regulations induced by PT-containing food were to suppress the expression of phosphoryl-transfer genes. The PT-containing diet probably alleviated DNA replication stress by its anti-ROS activity, which might result in less cellular damage^50–52^. So, the cell cycle can run at a relatively low rate without changing worms’ development and growth. It is not clear how (or even, if) phosphorothioate suppresses gene expressions at the DNA–RNA transcription level.

Since the top-ranking GO terms were dominated by down-regulated genes that had much lower *p*_*adj*_ values than up-regulated genes, we specifically checked the top-three GO terms corresponding to gene upregulation. Those top three GO terms in each of the three GO aspects were collagen trimer, ciliary part, and cell projection part in the ‘cell component’ class; neuropeptide signaling pathway, phototransduction, and detection of light stimulus in the ‘biological process’ class and structural constituent of cuticle, oxygen binding, and tetrapyrrole binding in the ‘molecular function’ class. Owing to the poor *p*_adj_-values, the top-ranking GO terms may change if only down-regulated genes were considered. Among them, the upregulation on neural signaling pathways may connect the high motility of the worms fed with PT-containing food, the oxygen and tetrapyrrole binding may relate to P450-like genes, and the phototransduction and detection of light stimulus (probably on cuticle and ciliary parts) may be related to ultraviolet sensitivity.

In the D12 samples, defense response, innate immune response, immune response, immune system process, and defense response to other organisms were the top five categories attributed to ‘biological process’ (Supplementary Data 4). Structural molecule activity, structural constituent of cuticle, carbohydrate binding, peptidase activity, acting on L-amino acid peptide activity, and protein dimerization activity were the top five categories in the ‘molecular function’ aspect. Immune response and protein dimerization were reduced, probably the PT-fed nematodes were aging more slowly and less aging-related immune response. The overall differences in the D12 samples were much smaller than those in the D4 samples, and no specific loci of the ‘cell component’ aspect was captured in the GO analysis of the D12 samples. The most significant change is phosphoryl-transfer terms (GO: 0031143/0018107/0018210/0006470 /0004721/0004672) that were downregulated at day 4 and upregulated at day 12, which may be related to the increasing motility at day 12 (see Supplementary Table 19).

### KEGG pathway enrichment of PT-feeding *C. elegans*

**Fig. 7:**
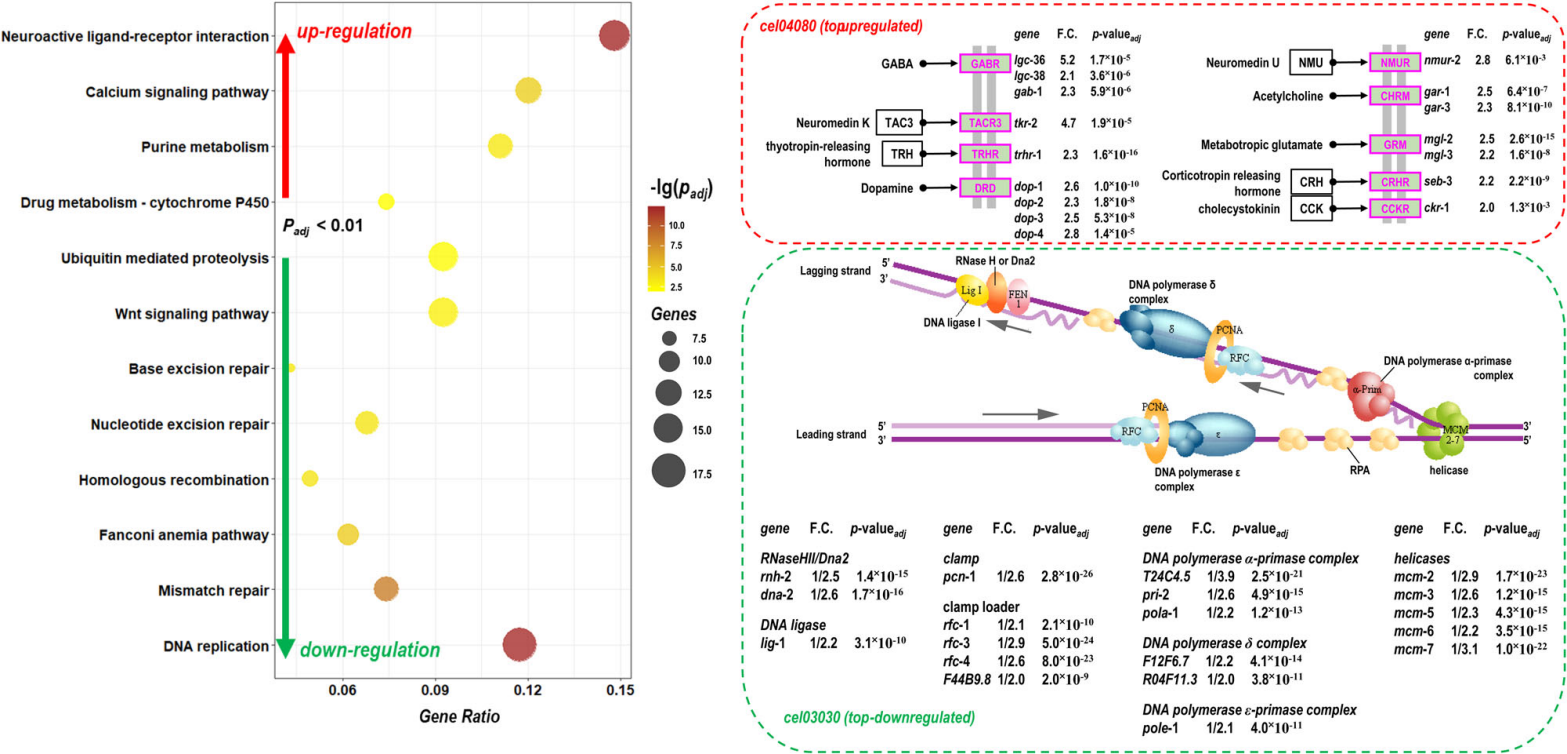
The KEGG pathway enrichment analysis of the differential transcriptomes for PT-fed *Caenorhabditis elegans*, using the S + /D4 vs S-/D4 sample. **a** The dotplot of enriched KEGG pathways with *p*_adj_ < 0.01, **b** the up-regulated gene expressions in the top-ranked neuroactive ligand-receptor interaction pathway (cel04080), **c** the down-regulated gene expressions in the top-ranked DNA replication pathway (cel03030).

The Kyoto encyclopedia of genes and genomes (KEGG) pathway enrichment analyses for DEGs of S+/D4 compared S-/D4 are shown in Fig. 7. Among the top-three regulated pathways, both the DNA repair (cel03030) and mismatch repair (cel03430) pathways were down-regulated (*p*_*adj*_ = 1.4 × 10^−8^ and 1.3 × 10^−6^), but neuroactive ligand-receptor interaction (cel04080) was up-regulated (*p*_*adj*_ = 9.6 × 10^−7^). They may be correlated with the PT-DNA self-repair mechanism and the high motility observed in the phenotypes.

As shown in Fig. 7b–c, the most dramatic changes in the top-ranked regulation pathways were in a range of 2 to 3-fold, except for *lgc*-36 (~5.2 fold), *tkr*-2 (~4.7 fold), T24C4.5 (~3.9 fold), and *mcm*-7 (~3.1 fold). *lgc*-36 is a protein-coding gene of the gamma-aminobutyric acid receptor (GARP, α-subunit), with corresponding transcript F07B10.5.1 (17/34 GAAC/GTTC, 6476 bp). *tkr*-2 is a tachykinin G protein-coupled receptor gene, with corresponding transcript C49A9.7.1 (21/31 GAAC/GTTC, 7823 bp). T24C4.5 coded DNA primase (54/56 GAAC/GTTC, 13349 bp). *mcm*-7 is a DNA replication licensing factor gene, with corresponding transcript F32D1.10 (20/22 GAAC/GTTC, 6609 bp). Six of seven *mcm* helicase gene expressions decreased by 2.6-fold on average, which may be connected to the phosphorothioate destabilization of the B-type DNA helix^3,6^. There are at least two possible reasons for the lowered DNA replication and mismatch repair genes expressions, one could be less oxidative DNA damage due to a relatively low ROS level, and another could be the higher flexibility of double and triple-stranded helices in helicases upon phosphorothioate involvement. Global regulation of transcripts with similar multiplicative changes suggests that phosphorothioate systemically targets gene networks, rather than specific genes.

The other up-regulated pathways included neuroactive ligand-receptor interaction, calcium signaling pathway, purine metabolism, drug metabolism - cytochrome P450, metabolism of xenobiotics by cytochrome P450, and glutathione metabolism in the D4 samples, while only biosynthesis of amino acids, cysteine, and methionine metabolism pathways were upregulated in the D12 samples (Supplementary Data 5–6). Basically, the KEGG enrichment pattern was consistent with the GO enrichment pattern concerning upregulation of neuroactivity.

Interestingly, glutathione metabolism was upregulated in the D4 samples and many glutathione S-transferase (GST) genes were induced, consistent with the differential gene expression in Fig. 4. This suggests that PT-containing food would change sulfur-related metabolism in organisms.

The other downregulated pathways were the fanconi anemia pathway, homologous recombination, nucleotide excision repair, base excision repair, Wnt signaling pathway, ubiquitin mediated proteolysis, biosynthesis of amino acids, and the TGF-beta signaling pathway in the D4 samples, while no specific pathways were meaningfully downregulated in the D12 samples (Supplementary Data 7).

## Discussion

**Fig. 8:**
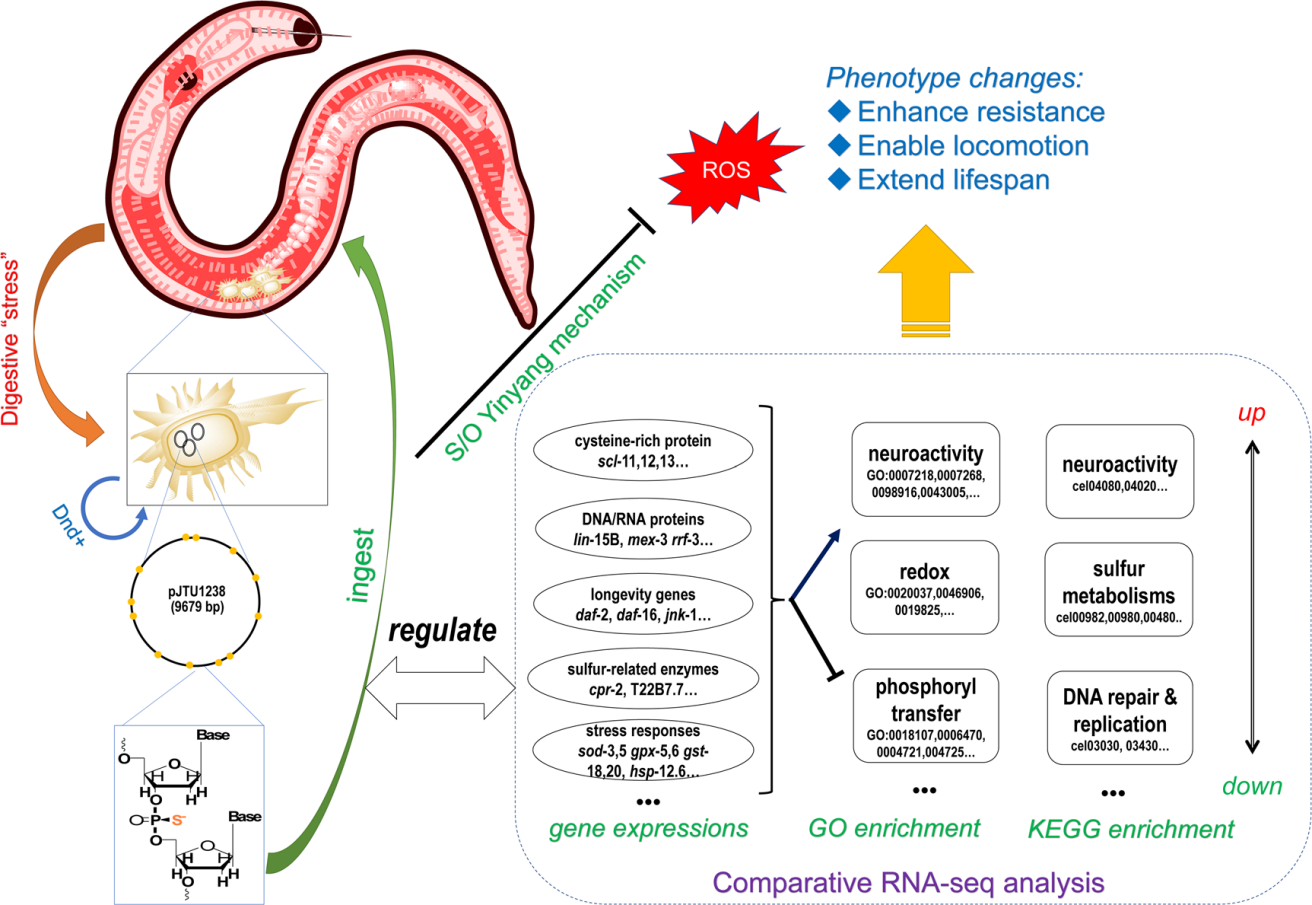
The plausible mechanism for the phenotype changes of *C. elegans* fed on PT-bacterial diet. The nematodes (pink-colored) uptakes *dnd*-gene bioengineering *E. coli* OP50 (brown-colored), in which plasmid and genome DNA undergo phosphorothioation by Dnd function proteins. The bacterial PT-DNA is thus ingested by the nematodes, converting ROS to reductive species via sulfur-oxygen one-atom chemistry. Simultaneously, the bacterial PT-DNA leads to globally interference on gene expressions of *scl*-11, 12, 13… The system-biological regulation is characterized by down-regulation of phosphoryl transfer GO terms and DNA repair/replication in KEGG enrichment, but up-regulation of neuroactivity. The overall effects enhance stress-resistance, enable locomotion, and extend lifespan.

The overall PT-containing bacterial food effects on *C. elegans* were integrated in Fig. 8. Basically, Dnd + bacteria brought their unique phosphorothioate materials into worms’ intestinal tract. Ultimately, bacteria were digested into small nutrient molecules, including phosphorothioate and their nucleotide oligomers. The long-term diet intake of phosphorothioate could help young nematodes to maintain a relatively low-level of ROS stress in their body, enabling higher locomotion and extending lifespan. The superior phenotypes could also contribute to the complicated regulation of gene expression by the phosphorothioate nutrients from the long-term Dnd + bacterial food, besides S/O anti-ROS activity.

The physiological roles of reactive oxygen species (ROS) can be quite complicated, and also divergent. ROS molecules are generated in aerobic metabolism, regulating many important cellular signaling transmissions; however, their over-generation causes oxidative damages, which is detrimental to cell function^53–55^. One side-effect of ROS accumulation during aerobic respiration is accelerated aging^56–58^. Traditionally, cellular antioxidants such as high-concentrations of vitamin C and free thiols (e.g., cysteine and glutathione) easily increase oxidative stress, namely prooxidativity^59–62^. The mild antioxidant phosphorothioate successfully avoids the prooxidative problem and is considered to be a high-quality antioxidant that only regulates the cellular ROS level to be within a healthy range^11,12^. In this study, we also observed a significant ROS-level decline measured by the H_2_O_2_ and peroxynitrite- specific probe, DCM. Even though *C. elegans* lacks a nitric oxide synthase gene, such that its ability to form peroxynitrite is questionable, bacterial nitric oxide is a nematode hormone^28^ and might be involved in peroxynitrite formation in the presence of superoxide.

Beyond the straightforward S/O anti-ROS mechanism, the regulation of gene expression of antioxidant enzymes such as superoxide dismutase, catalase, glutathione reductase, and glutathione peroxidase were stunning^63–66^. Previous bacterial transcriptome studies presented a paradox concerning whether the redox state of Dnd + bacteria was affected by PT-modification or not^1,18^. The bacterial investigations in literature have in part been inconclusive due to the dramatic difference of genetic backgrounds from *Streptomyces lividan*s to *E. coli* B7A, conflating gene-expression caused by PT-modification with that of the genetic baseline. In this work, the genetic background of the *C. elegans* N2 strain was identical for the two established groups of nematodes. Accordingly, gene-expression regulation became very stable and redox-related genes, like SODs and GSTs, were successfully identified.

Another interesting discovery was the potential enhancement of locomotion and neuroactivity by consumption of PT-containing food. To the best of our knowledge, support for the upregulation of neural signaling pathways was found for the first time, and affected worms retained the motility significantly longer. The molecular mechanism for phosphorothioate-neuroactivity has not been determined, but is a very promising avenue of future investigation. Particularly, the body size (an equivalent index of body weight) remained unchanged, making phosphorothioate a potential treatment for neurodegenerative diseases such as Alzheimer’s disease.

Indeed, phosphorothioate oligonucleotides have been long used in antisense drugs for clinical research since the 1970s^67–71^, for that phosphorothioate can slow down phosphoryl hydrolysis and thereby retain pharmacokinetics. Antisense drugs target many diseases, including neurodegenerative disorders, cancers, infections, and other diseases^68,70,71^. It is worth revisiting phosphorothioate’s cryptic function as anti-oxidant and neuro-activator.

Accordingly, the microbiomics roles of Dnd+ bacteria in human gut appear auspicious. Phosphorothioation was promoted under many stresses^20^, and thereby Dnd+ bacteria may increase the PT-modification frequency on their genomic materials. On the other hand, the chance for bridging P-O cleavage arises during the PS/PO conversion, which leads to genomic instability and bacterial death. Phosphorothioate release could help lower the surrounding ROS level for neighboring bacteria and the host organism. The different ROS level detected by the two fluorescent probes implies that ROS species were not evenly declining upon the PT-containing food, and in particular, hydrogen peroxide may decrease more significantly than other ROS species.

Finally, increased longevity could be one of the best discoveries from PT-fed *C. elegans*. Lifespan extension was confirmed by age-related lipofuscin attenuation. PT-diet upregulated 64.3% of Class I (4178 detected in this work) and downregulated 61.1% of Class II (3996 detected) in the over 18,000 *daf*-16 target genes (see Supplementary Fig. 19, also see the extending comparisons in Supplementary Figs. 20–21), indicating that PT-diet pro-longevity might share similar signaling pathways with *daf*-16 such as insulin/IGF-1 and dietary restriction pathways—the global regulation on gene expressions could be implemented by bacterial phosphorothioate-involving core sequence elements like DBE (*daf*-16 binding element) and DAE (*daf*-16 associate element)^72^. Multiple factors may influence prolonged life in *C. elegans*, including a lowered ROS level, suppressing phosphoryl transfer, alleviating DNA repair burden, and regulating redox enzyme and protein levels. The pro-longevity role of PT-diet is milder than that of *daf*-2/16 mutations, but is more practical for pharmaceutical application.

## Methods

### Strains and culture conditions

*Escherichia coli* OP50, which is used as the standard food for *C. elegans* cultivation, was grown on nematode growth medium (NGM) at 37 °C. Plasmids with *dndC-E* gene cluster were transformed to *E. coli* OP50 to construct a PT-DNA containing strain named *E. coli* OP50 (S^+^). Wild-type N2, short-lived mutant (*daf-16*)^73^ and long-lived mutant (*daf-2*)^74^ worm strains were received by professor Wang Da-Yong from Southeast University. Worms were maintained on NGM plates (1.7% agar, 2.5 mg/ml peptone, 25 mM NaCl, 50 mM KH_2_PO_4_, pH 6.0, 5 μg/ml cholesterol, 1 mM CaCl_2_, and 1 mM MgSO_4_) kept at 25 °C and spotted with *E. coli* OP50 or *E. coli* OP50 (S^+^) as the food source.

### Generalized assay preparation

Age-synchronization was accomplished by allowing ten young adult worms lay eggs on a fresh NGM plate with OP50 (S^−^) or OP50 (S^+^) for 4 h at 25°C. Adult worms were then removed and the plates were incubated at room temperature (25 ^o^C) for 3 days^75^.

### Measurement of Reactive Oxygen Species (ROS) in *Caenorhabditis elegans*

Worms were grown on NGM plates containing OP50 or OP50 (S^+^) bacteria, and then subjected to ROS assays. ROS formation in *C. elegans* was measured using the fluorescent probes 2,7-dichlorodihydrofluorescein diacetate (H_2_DCF-DA)^23–25^ and 2-(2-(4-(4,4,5,5-tetramethyl-1,3,2-dioxaborolan-2-yl)styryl)-4H-chromen-4-ylidene)malononitrile (DCM)^26,27^. The animals cultured at the appropriate time (1, 4, 8, or 12 days of adulthood) were collected into 1 mL phosphate-buffered saline (PBS) in Eppendorf tubes and washed 3 times with fresh PBS, then the nematodes were incubated with 50 μM H_2_DCF-DA and DCM for 60 min at room temperature separately, while the control was incubated with the same volume of DMSO. After that, animals were all washed 3 three times with fresh PBS and placed onto a slid with 3% agar plate containing 2 mM levamisole^59^. DCF Fluorescence intensity was measured at excitation and emission wavelengths of 488 and 510–560 nm, respectively, by means of laser scanning confocal microscopy (Leica & TCS SP8)^23–25^. The fluorescence intensity of DCM was measured by laser scanning confocal microscope (Leica & TCS SP8) at excitation and emission wavelengths of 488 and 630–750 nm, respectively^26,27^. The assay was performed in three independent trials, and at least 25 animals were used in each assay.

### Lipofuscin analysis

Worms were grown on NGM plates containing OP50 or OP50 (S^+^) bacteria, and then subjected to lipofuscin analysis. The autofluorescence of intestinal lipofuscin was measured as an index of senescence at day 1, 4, 8, and 12 of adulthood. Randomly selected worms from the plates with OP50 and OP50 (S^+^) were washed three times with M9 buffer. Worms were then placed onto a 3% agar plate containing 2 mM levamisole. Lipofuscin autofluorescence was detected by laser scanning confocal microscope at excitation and emission wavelengths of 478 and 550–600 nm, respectively (Leica & TCS SP8)^29^. The assay was performed in three independent trials and at least 20 animals were used in each assay.

### Measurement of locomotion

Synchronized three-day-old worms were placed on mNGM plates covered with lawns of OP50 and OP50 (S^+^). The motility of the worms was then examined using the scoring method described in previous studies^33^. Briefly, worms were placed on mNGM plates supplemented with 0.1 mM of FUdR (2′-deoxy-5-fluorouridine) and covered with lawns of OP50 and OP50 (S^+^) classified as class “a” when they exhibited spontaneous movement or vigorous locomotion in response to prodding; class “b” worms were those that did not move unless prodded or appeared to have uncoordinated movement; and class “c” worms were those that moved only their head and/or tail in response to prodding. Dead worms were classified as class “d”. Three independent replicates were tested, and the merged data were statistically analyzed and presented.

### Lifespan assay

Age-synchronization was accomplished as described above^34^. >90 worms were randomly selected from both groups and transferred to three fresh NGM plates with live OP50 or OP50 (S^+^), correspondingly, and supplemented with 0.1 mM of FUdR to avoid progeny. The animals were transferred daily to fresh plates for the first four days, and thereafter, they were transferred every other day until all nematodes were dead^74^. Animals were scored daily by gentle prodding with a platinum wire^76^. Those animals that failed to respond to a gentle touch with a sterilized platinum wire were scored as dead^75,76^. Animals that crawled off the plate or died from internal hatching were censored. Worm survival was calculated by the Kaplan–Meier method, and differences in survival were analyzed using the log-rank test. All experiments were performed at 25 °C, except for the heat shock treatment.

### Measurement of body size

Age-synchronized nematodes were prepared as describe above, then grown on NGM plates covered with lawns of OP50 or OP50 (S^+^) until the late larval stage, L4 (3 days). >90 adult worms were placed on 3 fresh plates with OP50 or OP50 (S^+^), and supplemented with 0.1 mM of FUdR to avoid progeny. The plates were incubated at 25 °C, and the body size of the live worms was measured every day until the worms reached the age of 10 days. Images of nematodes were taken with a stereoscopic microscope (Shanghai Bimu & XTL-3400E) equipped with a camera and analyzed by ImageJ software. The areas of a worm’s projection were used as an index of its body size.

### Measurement of brood size

Age-synchronized nematodes were prepared as if for a lifespan assay, then grown on NGM plates covered with lawns of OP50 or OP50 (S^+^) until the late larval stage, L4 (3 days). Worms were then transferred to fresh plates, one animal per plate, with *N* = 8 animals per group. Thereafter, animals were transferred every 24 h to fresh plates with OP50 or OP50 (S^+^) bacteria until the end of the reproductive period^77^. The total number of progenies that grew up from each animal was counted, and the number of progenies for each concentration were averaged.

### Stress resistance assays

Worms were grown on NGM plates containing OP50 or OP50 (S^+^) bacteria for a long time, and then subjected to stress resistance assays. Stress resistance assay were performed as previous described^34^. For oxidative stress, the 7-day old worms were transferred to a 24-well plate containing 30 mM paraquat (Sigma-Aldrich)^33^. The animals were monitored every 12 h and were scored as dead when they did not move in the liquid medium. These assays were each performed in three independent trials. Each group included at least 30 animals. For heat shock assays, the worms were transferred to an incubator set to 35 °C for 6 h and then scored for viability every hour^33^. Similarly, for the heavy metal stress assay, 3-day-old worms were transferred to a 24-well plate containing 10 mM K_2_Cr_2_O_7_^34^. The animals were monitored every 12 h and were scored as dead when they did not move in the liquid medium. For the ultraviolet (UV) irradiation assay, 3-day-old animals were transferred to bacteria-free NGM plates and UV-irradiated at 600 J/m^2^ with an instrument equipped with a 5-watt 180-nm UV light bulb^34^. After UV irradiation, animals were transferred to fresh NGM plates with OP50 or OP50 (S^+^) and monitored daily for survival by gentle prodding with a platinum wire.

For stress resistance assays of animals fed different antioxidants, worms were grown from the age of 3 days on NGM plates containing antioxidants (DETP, vitamin C, or NAC) or on plates not containing antioxidants, and then subjected to stress resistance assays. For oxidative stress, 7-day old worms were transferred to a 24-well plate containing 30 mM paraquat (Sigma-Aldrich)^33^. The animals were monitored every 12 h and were scored as dead when they did not move in the liquid medium. These assays were each performed in three independent trials. Each group included at least 30 animals. Similarly, for the heavy metal stress assay, 3-day-old worms were transferred to a 24-well plate containing 10 mM K_2_Cr_2_O_7_. The animals were monitored every 12 h and were scored as dead when they did not move in the liquid medium.

### RNA sequencing and data analysis

Age-synchronized nematodes were prepared as described above. Worms were grown on S medium with OP50 or OP50 (S^+^) bacteria. Three biological replicates of animals at 4 days and 12 days after the L4 stage were used for RNA sequencing. A total of 3 μg RNA per sample was used as input material for the RNA sample preparations. RNA quality was evaluated on a Nano Photometer spectrophotometer (IMPLEN, Munich, Germany), and the RNA integrity characterized using a Bioanalyzer 2100 (Agilent, Santa Clara, CA). Sequencing libraries were prepared following the protocol of the NEBNext Ultra RNA library Prep Kit (NEB) and sequenced on an Illumina HiSeq X Ten platform in the paired‐end mode (2 × 150 bp) through the service provided by Bionova. RNA-seq reads were aligned to the *C. elegans* reference genome (ws235) using HISAT2 (v2.0.5^78^) with default parameters. Gene‐level read counts were calculated using HTSeq2 (v0.6.1p1^79^) based on the Ensemble gene annotation v85. DEseq2 (v1.18.1^80^) was used for data normalization.

Statistical analysis of differential expression was performed using the *nbinomWaldTest* in the DESeq2 package. Genes with an adjusted *p*‐value < 0.01 and |log2Foldchange| > 1 were defined as differentially expressed genes (DEGs).

### Gene quantification through real-time PCR

Age-synchronized nematodes were prepared as described above. Worms were grown on S medium with OP50 and OP50 (S^+^) bacteria. Three biological replicates of animals at 4 days and 12 days after the L4 stage were used for the RNA isolation. The first-strand cDNA synthesis was then performed from the total RNA with the Maxima H Minus M-MuLV reverse transcriptase kit (Sangon Biotech, China). Expression levels of genes were measured with internal control of *bmp-3*. Real-time PCR was carried out by the 2 × SG Fast qPCR Master Mix Kit (Sangon Biotech, China) on Analytikjena & qTOWER3G touch (Analytikjena, Germany). Three independent trials were performed, and the relative mRNA quantification of the desired genes was analyzed by the comparative CT (ΔΔ CT) method^46^. The qRT-PCR primers were listed in Supplementary Table 20.

### Statistics and reproducibility

All experiments were performed at least three times. For in vivo experiments in *C. elegans*, All the results were expressed as (mean value ± standard deviation). The statistical significance was calculated with the Student’s *t* test and log-rank test using GraphPad Prism. *p* values < 0.05 were considered to be significant.

### Reporting summary

Further information on research design is available in the Nature Research Reporting Summary linked to this article.

## Supplementary information

Supplementary Information

Description of Additional Supplementary Files

Supplementary Data 1

Supplementary Data 2

Supplementary Data 3

Supplementary Data 4

Supplementary Data 5

Supplementary Data 6

Supplementary Data 7

Supplementary Data 8

Reporting Summary

## Data Availability

RNAseq data that support the findings of this study have been deposited in the Sequence Read Archive (SRA) at NCBI with the accession codes SRR16378842, SRR16378843, SRR16378844, and SRR16378845 (https://www.ncbi.nlm.nih.gov/sra/PRJNA772010). The authors declare that the other data supporting the findings of this study are available within the paper and its supplementary information files. Source data for figures are provided with the paper as Supplementary Data 1–8. The raw figure data that supporting Supplementary Figs. 1–3 are available in FIGSHARE with the identifiers 10.6084/m9.figshare.16909399.v1 (all the panels in Supplementary Fig. 1), 10.6084/m9.figshare.16909417.v1 (all the panels in Supplementary Fig. 2), 10.6084/m9.figshare.16909426.v1 (all the panels in Supplementary Fig. 3).
