# Author Correction: Phosphorothioate-DNA bacterial diet reduces the ROS levels in *C. elegans* while improving locomotion and longevity

**DOI:** 10.1038/s42003-021-02932-2

**Published:** 2021-12-14

**Authors:** Qiang Huang, Ruohan Li, Tao Yi, Fengsong Cong, Dayong Wang, Zixin Deng, Yi-Lei Zhao

**Affiliations:** 1 State Key Laboratory of Microbial Metabolism, Joint International Research Laboratory of Metabolic & Developmental Sciences, Department of Bioinformatics and Biostatistics, School of Life Sciences and Biotechnology, Shanghai Jiao Tong University, Shanghai, 200240 China; 2 Department of Chemistry, Fudan University, 2005 Songhu Road, Shanghai, 200438 China; 3 College of Chemistry, Chemical Engineering and Biotechnology, Donghua University, Shanghai, 201620 China; 4 National Experimental Teaching Center, School of Life Sciences and Biotechnology, Shanghai Jiao Tong University, Shanghai, 200240 P.R. China; 5 Key Laboratory of Environmental Medicine Engineering in Ministry of Education, Medical School, Southeast University, Nanjing, 210009 China

**Keywords:** Ageing, Bacterial host response

Correction to: *Communications Biology* 10.1038/s42003-021-02863-y, published online 25 November 2021.

In the original published version of the Article, the abstract incorrectly listed *hsp-*12.8 as one of the differentially-expressed stress response genes. The correct gene symbol is *hsp-*12.6. The error has been corrected in the HTML and PDF versions of the Article.

