# Supplementary material for: Phosphorothioate-DNA bacterial diet reduces the ROS levels in C. elegans while improving locomotion and longevity
Source: Commun Biol. 2021 Nov 25;4:1335. doi: 10.1038/s42003-021-02863-y (PMC8617147; doi:10.1038/s42003-021-02863-y)
Supplement: Supplementary file 1 — Supplementary Information [file 42003_2021_2863_MOESM1_ESM.pdf]

Supplementary Information

Phosphorothioate-DNA bacterial diet reduces the ROS levels in *C. elegans* while improving locomotion and longevity

*Qiang Huang[1], Ruohan Li,[2] Tao Yi,[2,3] Fengsong Cong,[4] Dayong Wang,[5] Zixin Deng,[1] Yi-Lei Zhao[1]**

[1]State Key Laboratory of Microbial Metabolism, Joint International Research Laboratory of Metabolic & Developmental Sciences, Department of Bioinformatics and Biostatistics, School of Life Sciences and Biotechnology, Shanghai Jiao Tong University, Shanghai, 200240, China.

[2.]Department of Chemistry, Fudan University, 2005 Songhu Road, Shanghai, 200438 China.

[3.]College of Chemistry, Chemical Engineering and Biotechnology, Donghua University, Shanghai, 201620 China

[4]National Experimental Teaching Center, School of Life Sciences and Biotechnology, Shanghai Jiao Tong University, Shanghai 200240, P.R. China.

[5]Key Laboratory of Environmental Medicine Engineering in Ministry of Education, Medical School, Southeast University, Nanjing, 210009, China.

*Email: yileizhao@sjtu.edu.cn

Contents

Supp. Fig. 1: DCF fluorescence imaging in *C. elegans*……..…………….…………....…………...…S3
Supp. Fig. 2: DCM fluorescence imaging in *C. elegans*…………………………..……....…………..S4
Supp. Fig. 3: Imaging of lipofuscin accumulation of *C. elegans*..……....…..…………………....…..S5
Supp. Fig. 4: locomotion during aging in *C. elegans*….………………….....……….……….....…...…S6
Supp. Fig. 5: The longevity of the two types of mutants……………..……………....………...……....S7
Supp. Fig. 6: Comparison of N2 C. elegans fed on alive and heat-killed bacteria...……………………S8
Supp. Fig. 7: Comparison of daf-2 and daf-16 C. elegans fed on alive and heat-killed bacteria………S9
Supp. Fig. 8: Susceptibility of DETP-fed *C. elegans* on paraquat and Cr^{6+} induced stress……………..S10
Supp. Fig. 9: PT-modified bacteria and PT-modified DNA under ultraviolet radiation……..…...…...S11
Supp. Fig. 10: FPKM density distribution of *C. elegans*…….………...…………………….……...S12
Supp. Fig. 11: Upregulated genes correlated with the temporal pattern of anti-ROS response………..S13
Supp. Fig. 12: Downregulated genes correlated with the temporal pattern of anti-ROS response……S14
Supp. Fig. 13: Upregulated genes correlated with the temporal pattern of motility………...…………S15
Supp. Fig. 14: Downregulated genes correlated with the temporal pattern of motility……….....……....S16
Supp. Fig. 15: Upregulated genes correlated with PT-diet in both the D4 and D12 samples....….……S17
Supp. Fig. 16: Downregulated genes correlated with PT-diet in both the D4 and D12 samples...……S18
Supp. Fig. 17: Correlation of gene expressions measured by the qRT-PCR and RNAseq……………S19
Supp. Fig. 18: Barplots of gene expressions measured by the qRT-PCR and RNAseq………….……S20
Supp. Fig. 19: Comparison of PT-diet gene regulation and daf-16 target genes…………...…..……S21
Supp. Fig. 20: Comparison with the genes specific in daf-16A and daf-16F isoforms……………..…S22
Supp. Fig. 21: Heatmap of the PT-diet regulation overlapped with daf-16 cofactors SWI/SNF..…..…S23
Supp. Table 1: Summary of relative fluorescence units of *C. elegans* under normal condition………S24
Supp. Table 2: Summary of lifespan of *C. elegans* under normal condition………….……………....S25
Supp. Table 3: Summary of lifespan of N2 *C. elegans* fed on alive and dead bacteria………………S26
Supp. Table 4: Summary of lifespan of daf-2 and daf-16 *C. elegans* fed on alive and dead bacteria…S27
Supp. Table 5: Summary of lifespan of *C. elegans* under stress……………..………………………..S28
Supp. Table 6: Summary of lifespan of *C. elegans* fed on antioxidants under stress...…………..………S29
Supp. Table 7: The number of different express genes in *C. elegans*…………..……………………..S30
Supp. Table 8: Top-100 upregulated genes correlated with the temporality of ROS-response………S31
Supp. Table 9: Top-100 downregulated genes correlated with the temporality of ROS-response……S32
Supp. Table 10: Top-100 upregulated genes correlated with the temporality of motility…………..……S33
Supp. Table 11: Downregulated genes correlated with the temporality of motility …………………S34
Supp. Table 12: Upregulated genes in both the D4(S+) and D12(S+) samples……………….…..……..S35
Supp. Table 13: Top-100 upregulated genes in the D4(S+) samples……………………………...……...S36
Supp. Table 14: Top-100 downregulated genes in the D4(S+) samples…………………………..……...S37
Supp. Table 15: Regulatory profiles of target genes related to daf-16/hsf-1/skn-1………………...…S38
Supp. Table 16: Stress response genes of *C. elegans* were induced by PT………………………..…...S39
Supp. Table 17: Aging-related genes regulated by PT-diet…………………………………..…..……S40
Supp. Table 18: Comparison between qRT-PCR and RNAseq measurements……….………..……..S41
Supp. Table 19: Top-ranking enriched GO terms in the D12 samples………………….……………..S42
Supp. Table 20: qRT-PCR primers……………………………………….……………….……………..S43
Supp. References …….…...…………………………………………………….…………….....S44-45

Supplementary Figure 1

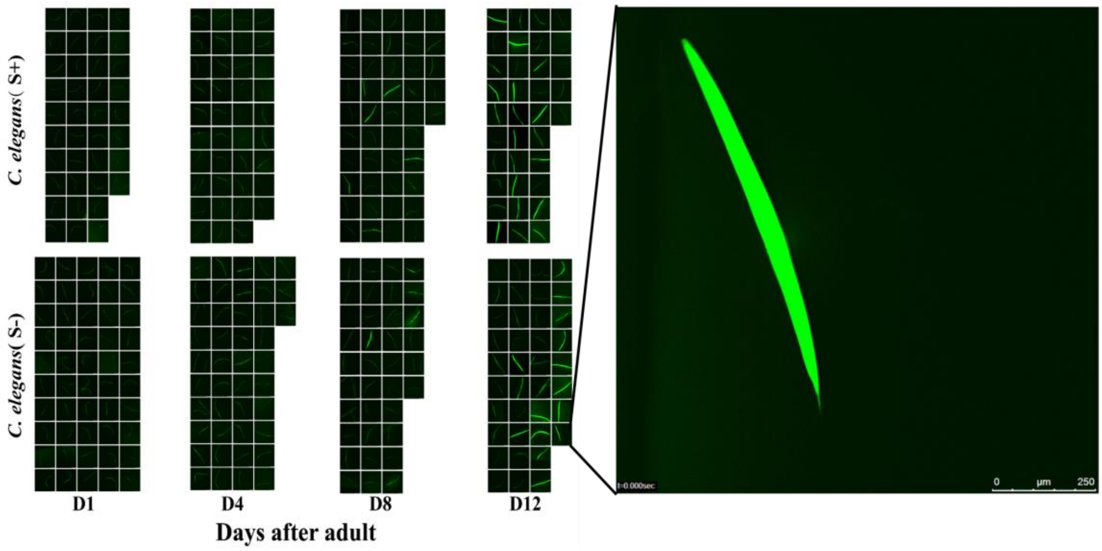

Supplementary Figure 1. DCF fluorescence imaging in *C. elegans* fed with OP50 (S^+) and OP50 (S^-) during aging. The fluorescence pictures show the total ROS accumulation in worms fed with OP50 (S^+) and OP50 cultured in 22 ºC at 1, 4, 8, 12 d after L4 larva. OP50 (S^+), n > 35, OP50 (S^-), n > 36. Right: a zoom view for the last panel in the grouped picture.

Supplementary Figure 2

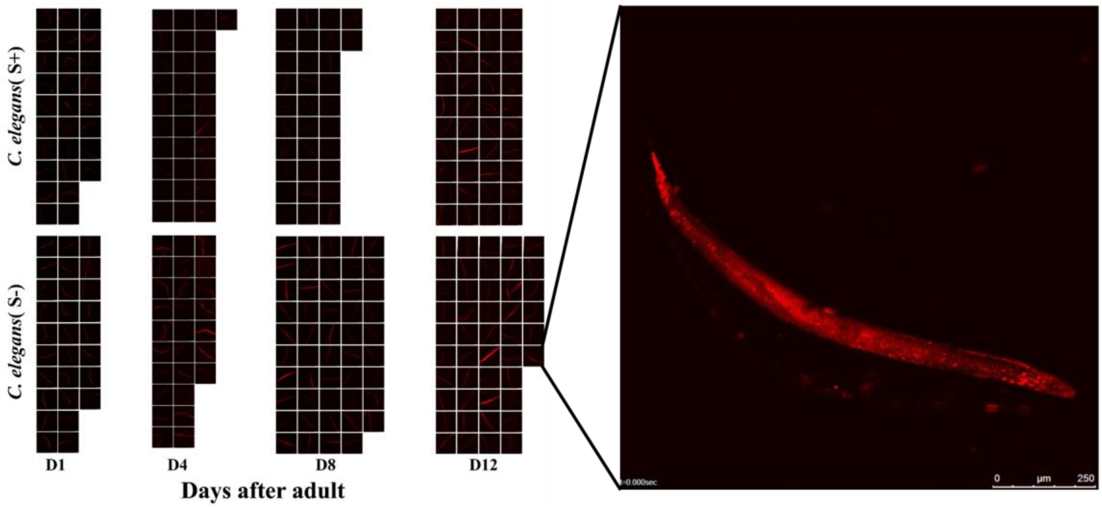

Supplementary Figure 2. DCM fluorescence imaging in *C. elegans* fed with OP50 (S^+) and OP50 (S^-) during aging. The fluorescence pictures show the peroxynitrite accumulation in worms fed with OP50 (S^+) and OP50 cultured in 22 ºC at 1, 4, 8, 12 d after L4 larva. OP50 (S^+), n > 28, OP50 (S^-), n > 27

Supplementary Figure 3

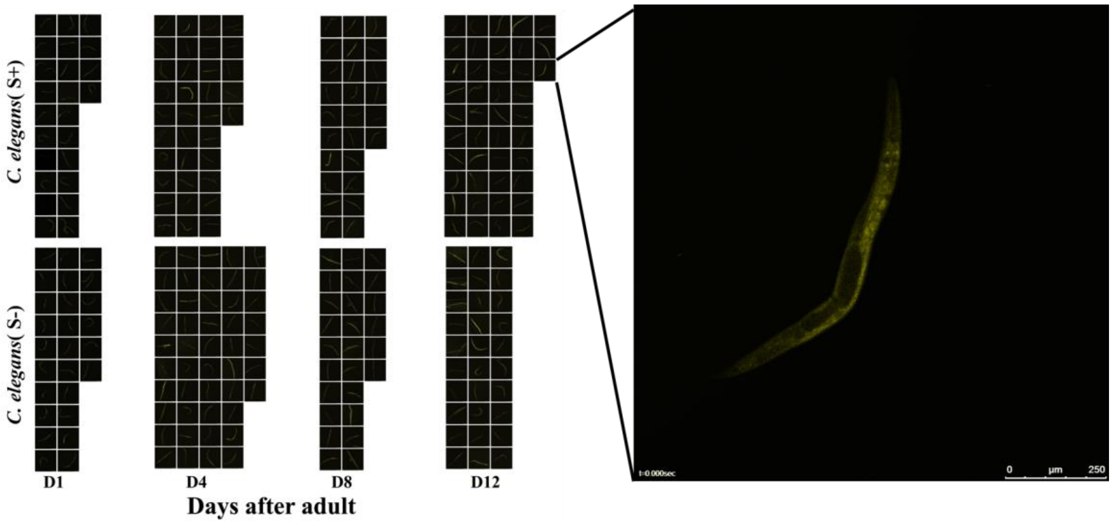

Supplementary Figure 3. Imaging of lipofuscin accumulation of *C. elegans* (N2) fed with OP50 (S^+) and OP50. The autofluorescence pictures show the lipofuscin accumulation in worms fed with OP50 (S^+) and OP50 cultured in 22 ºC at 1, 4, 8, 12 d after L4 larva. OP50 (S^+), n > 24, OP50 (S^-), n > 26

Supplementary Figure 4

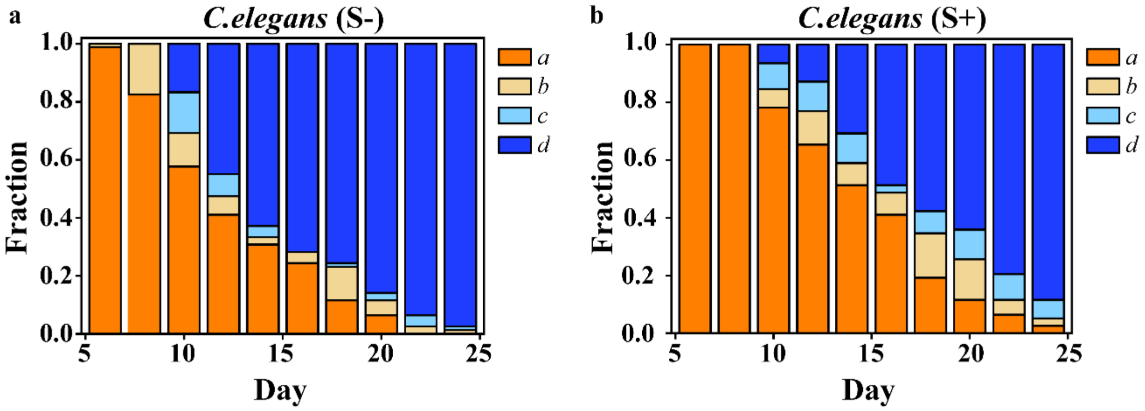

Supplementary Figure 4. OP50 (S$^+$) improved locomotion during aging in *C. elegans*. A-B, Locomotory activity of *C. elegans* fed OP50 (A) or OP50 (S$^+$) (B). Animals were grouped into the following four classes based on their locomotion: class a, robust, coordinated sinusoidal locomotion (orange bars); class b, uncoordinated and/or sluggish movement (light orange bars); class c, no forward or backward movement, but head movements or shuddering in response to prodding (light blue bars); and class d, dead animals (blue bars). The frequency of each class at the indicated time point is indicated. n > 70 for each assay.

Supplementary Figure 5

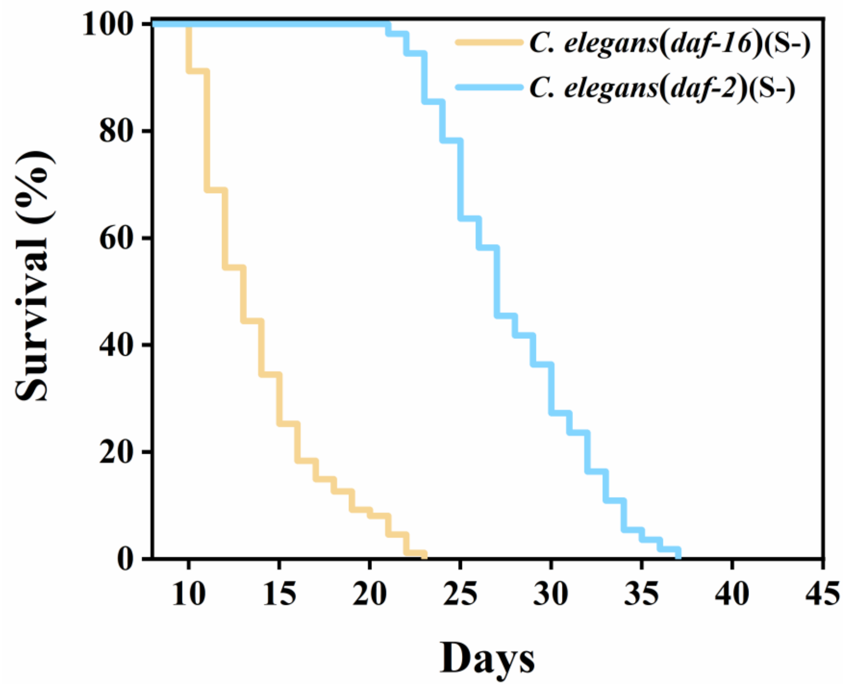

Supplementary Figure 5. The longevity of the two types of mutants, using the nominal age of 3 days at the starting point (*daf-2* has a longer lifespan. p-value < 0.001, n_{N2} = 132, $n_{daf\text{-}2}$= 117, *daf-16* has a shorter lifespan. p-value < 0.01, n_{N2} = 132, $n_{daf\text{-}16}$= 114).

Supplementary Figure 6

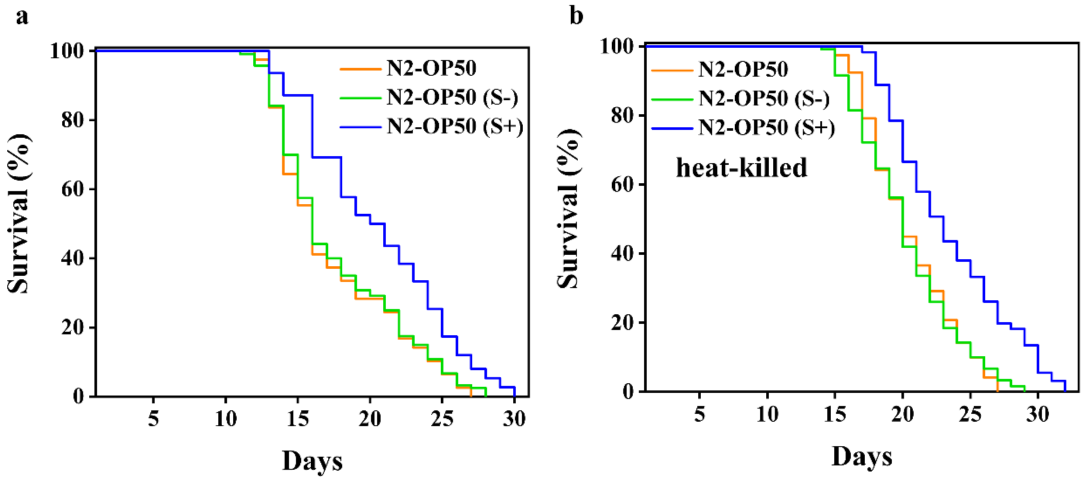

Supplementary Figure 6. Survival curves of *C. elegans* (wild type N2 strains) fed on (A) alive (*C. elegans* fed on S+ has a longer lifespan. *p*-value < 0.001) and (B) heat-killed OP50s (*C. elegans* fed on S+ has a longer lifespan. *p*-value < 0.001). (nematode numbers in experiment: alive bacterial diet group, 124 fed on wild type OP50 *E. coli*, 132 on PT-engineering OP50 (S+), and 120 on *dndC*-disrupted bioengineering OP50 (S-); heat-killed OP50 diet group, 128 fed on wild type OP50 *E. coli*, 127 on PT-engineering OP50 (S+), and 119 on *dndC*-disrupted bioengineering OP50 (S-)). The statistical significance was calculated with log-rank test.

Supplementary Figure 7

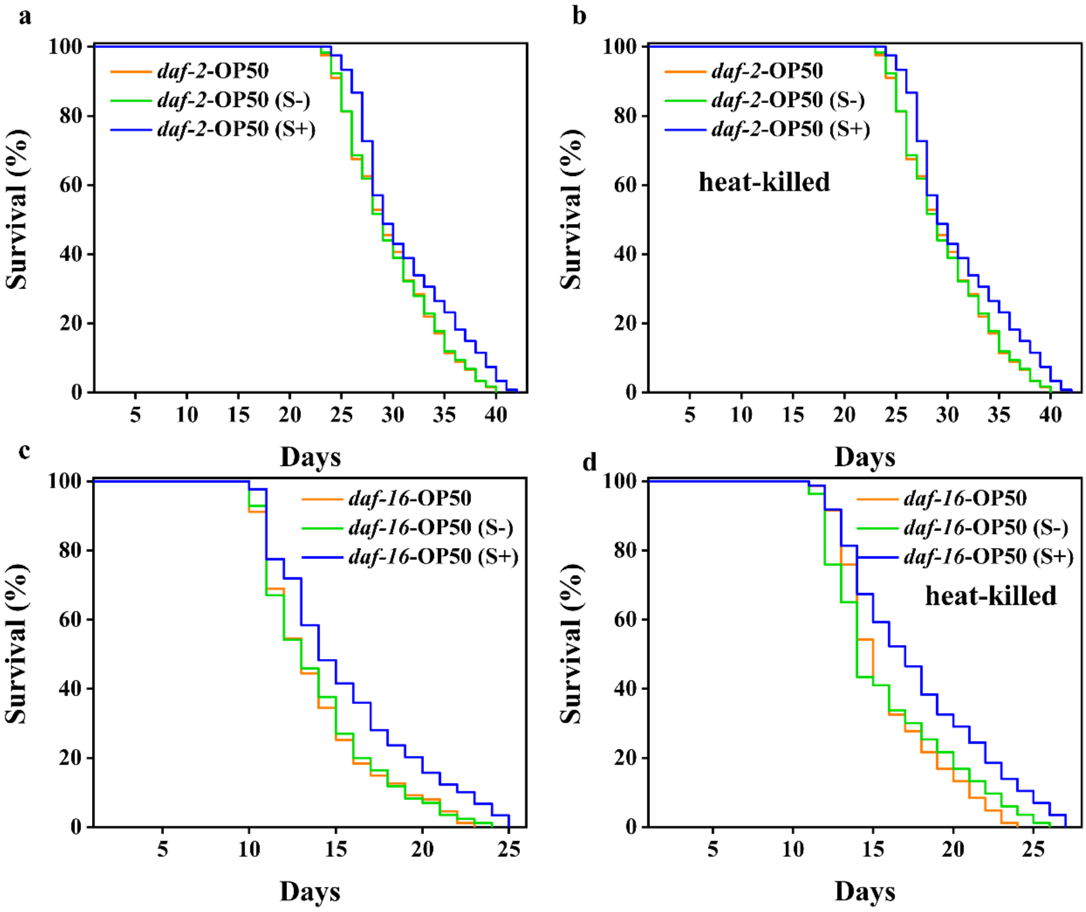

Supplementary Figure 7. Survival curves of the two genotypes of nematodes fed with alive and heat-killed OP50 bacteria (OP50, OP50 (S-), and OP50 (S+)). (a) long-lived *daf*-2 mutant fed on alive bacteria (*C. elegans* fed on S+ has a longer lifespan. *p*-value = 0.01), (b) *daf*-2 fed on dead bacteria (*C. elegans* fed on S+ has a longer lifespan. *p*-value = 0.02), (c) short-lived *daf*-16 fed on alive bacteria (*C. elegans* fed on S+ has a longer lifespan. *p*-value = 0.03), and (d) *daf*-16 fed on dead bacteria (*C. elegans* fed on S+ has a longer lifespan. *p*-value = 0.003). The statistical significance was calculated with log-rank test.

Supplementary Figure 8

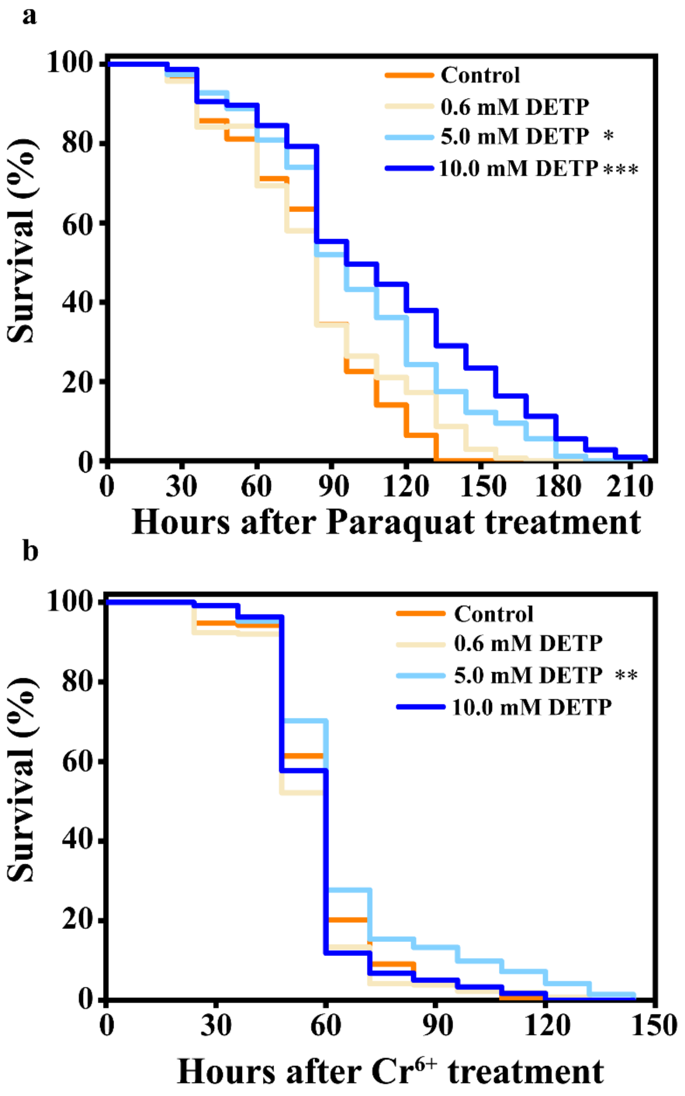

Supplementary Figure 8. Susceptibility of DETP-fed *C. elegans* (N2) on paraquat and Cr^{6+} induced stress. Worms were pre-treated with different concentration of DETP and and then subjected to 30 mM paraquat or 10 mM $K_2Cr_2O_7$. Differences in survival were analyzed using the log-rank test. *Statistical significance at $p < 0.05$ vs. control-fed group; **Statistical significance at $p < 0.01$ vs. control-fed group; ***Statistical significance at $p < 0.001$ vs. control-fed group.

Supplementary Figure 9

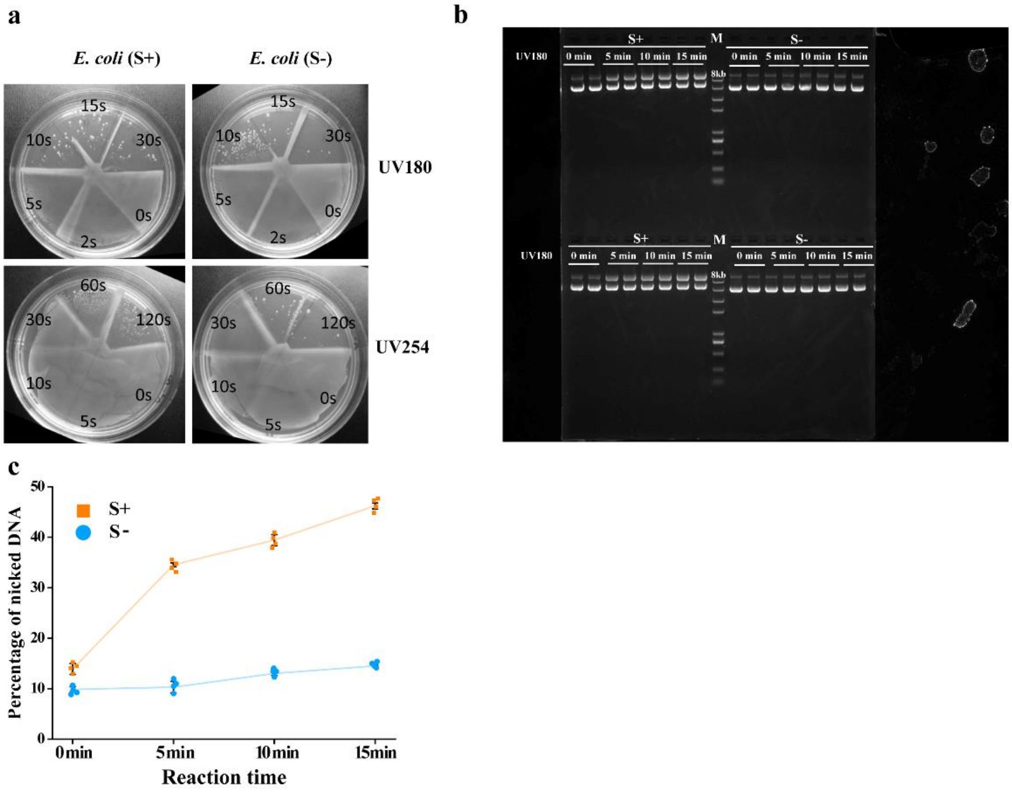

Supplementary Figure 9. Detection of the survival rate of PT-modified bacteria (a) and the damage level of PT-modified DNA under ultraviolet radiation (b) and (c). Central lines represent the mean values, and error bars note the standard deviation.

Supplementary Figure 10

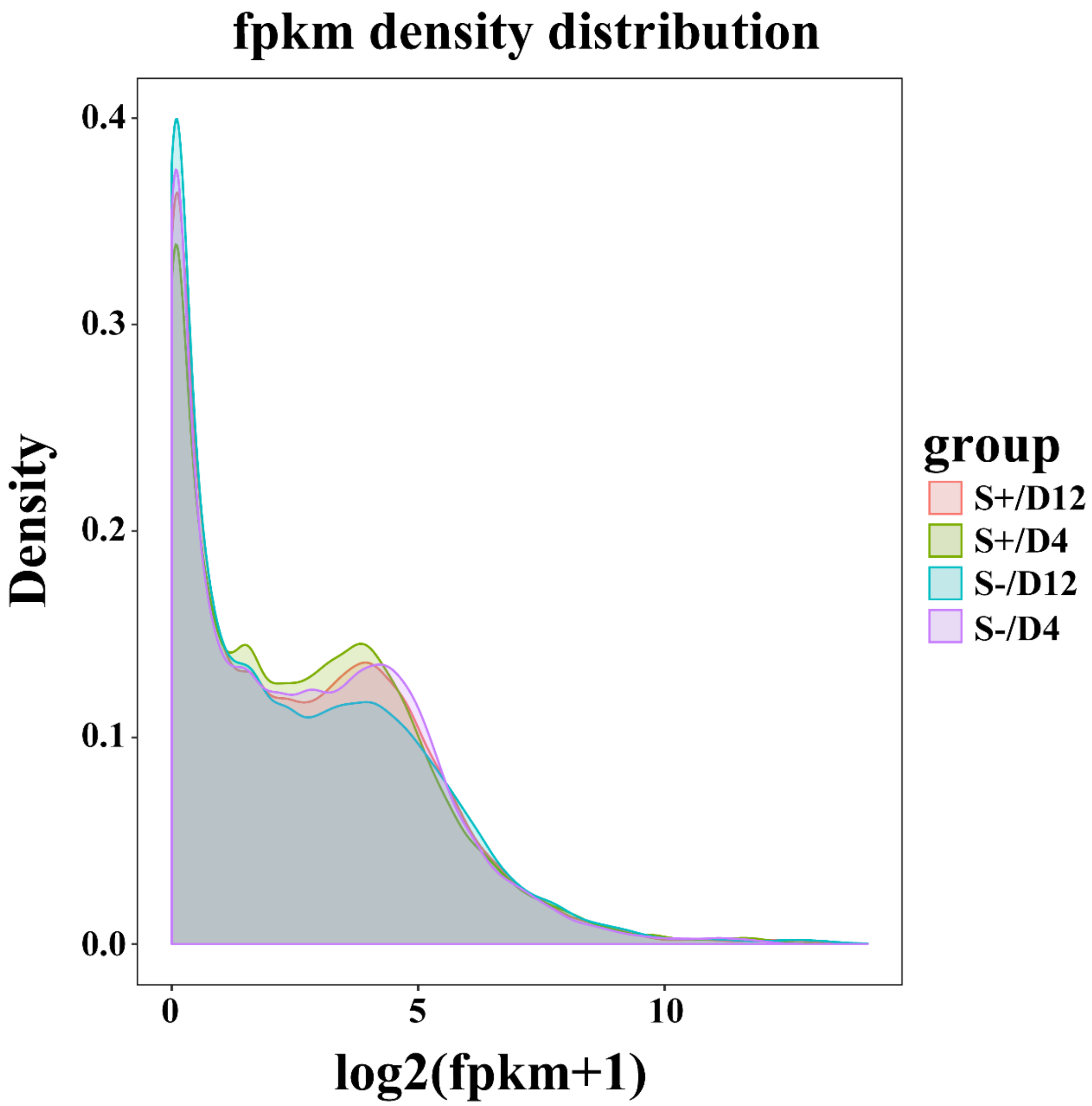

Supplementary Figure 10. The fpkm density distribution of *C. elegans* (N2) fed with PT-containing OP50 or non-PT-containing OP50 at different stage

Supplementary Figure 11

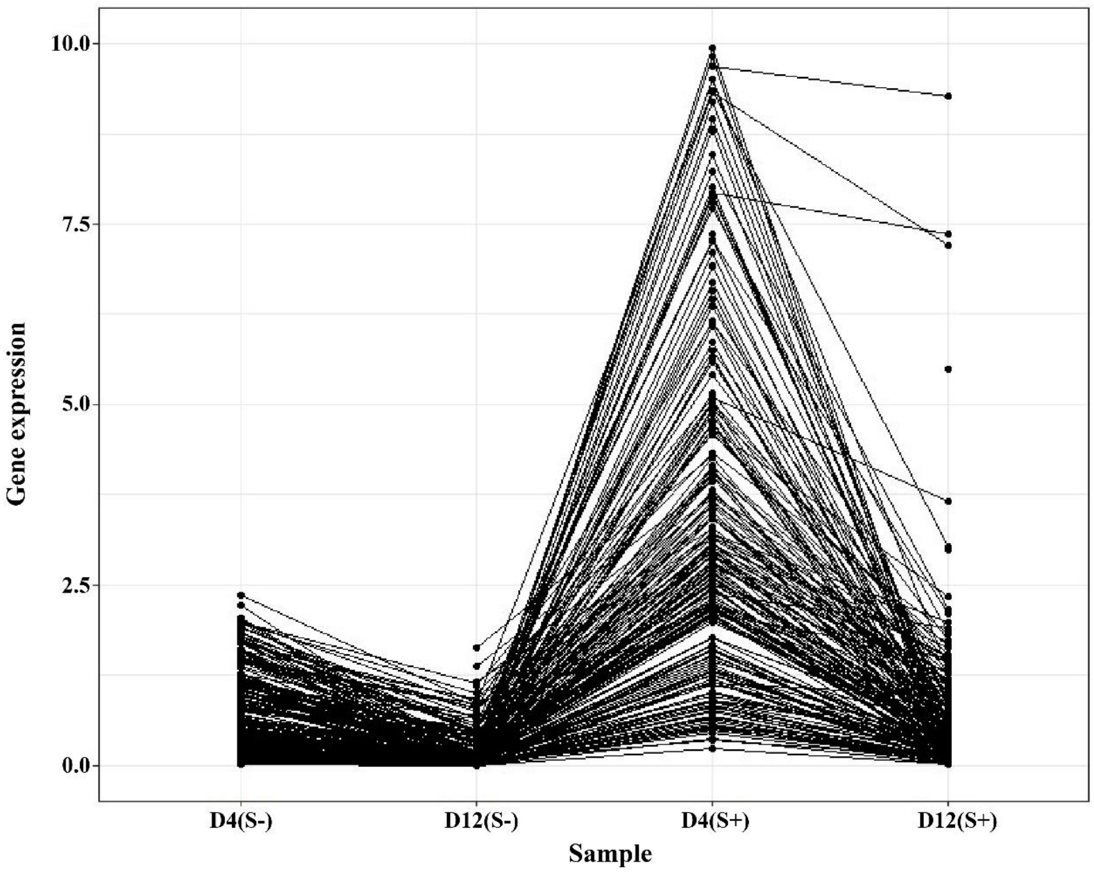

Supplementary Figure 11. The upregulated genes correlated with the anti-ROS response in hypothetical chronology.

Supplementary Figure 12

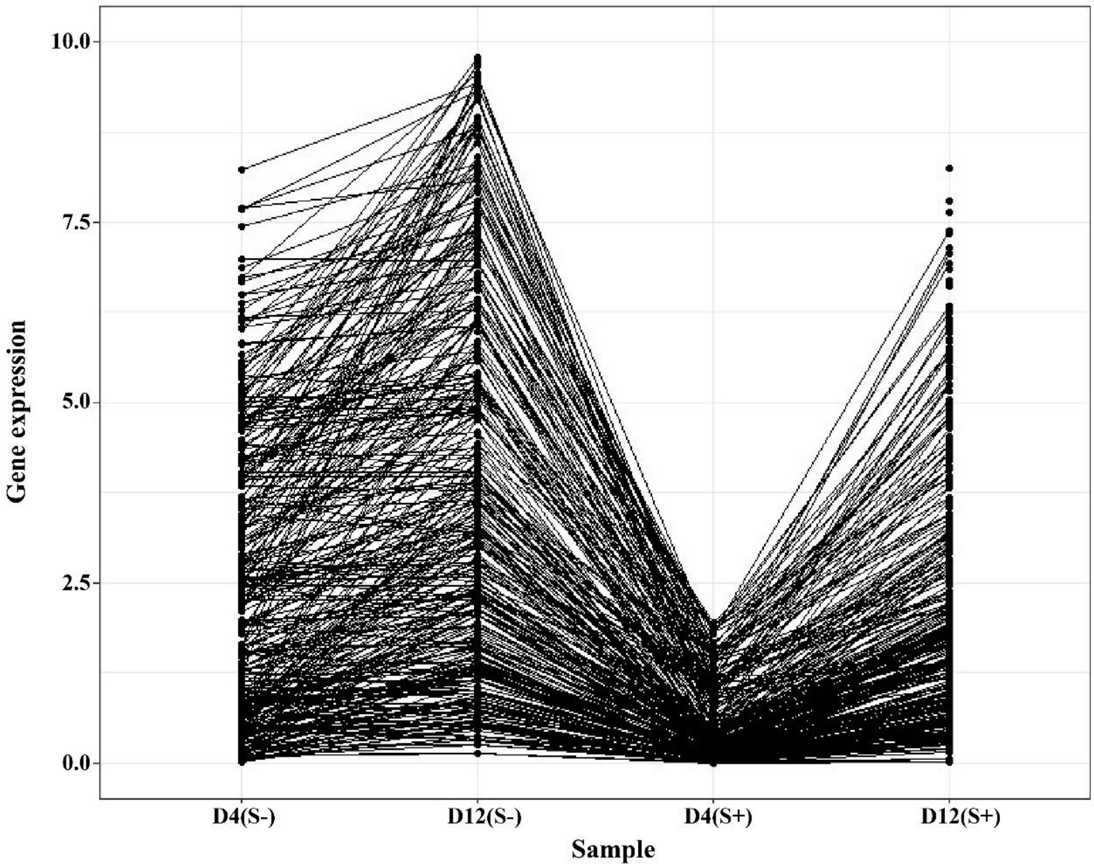

Supplementary Figure 12. The downregulated gene correlated with the anti-ROS response in hypothetical chronology.

Supplementary Figure 13

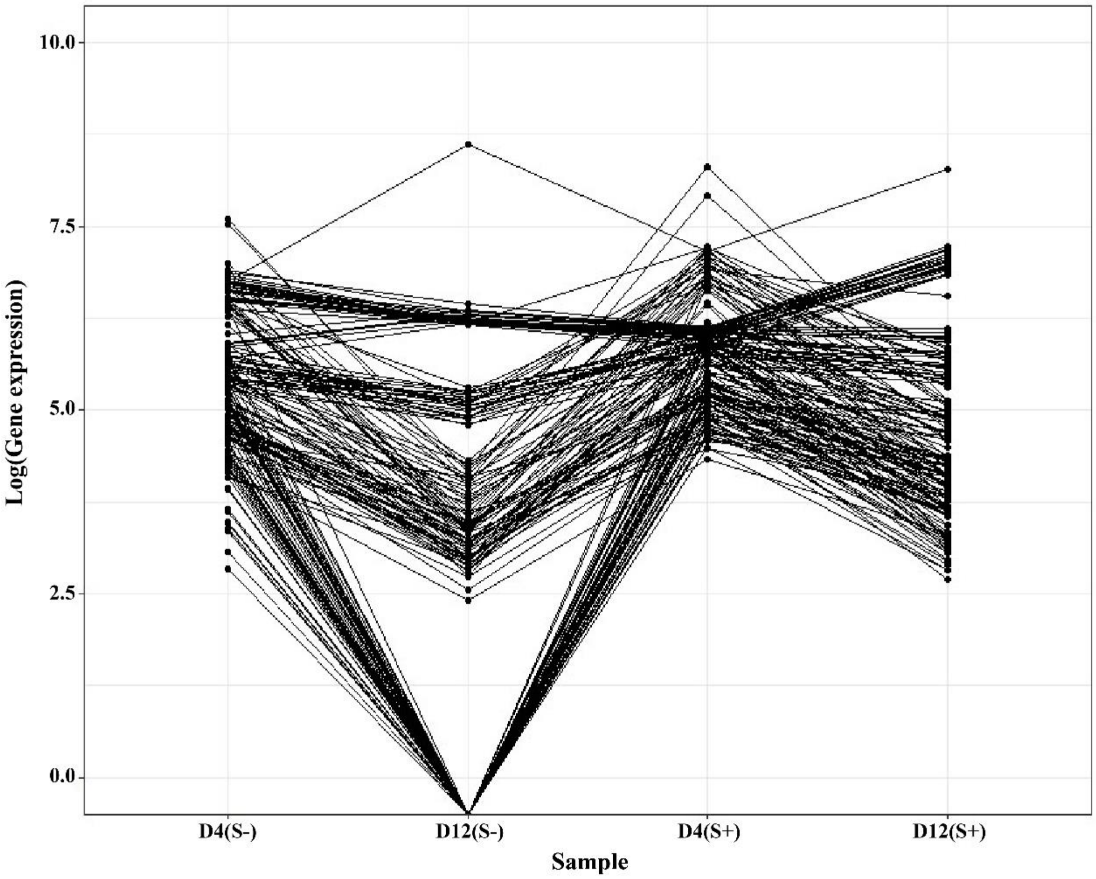

Supplementary Figure 13. The upregulated gene correlated with the motility in hypothetical chronology.

Supplementary Figure 14

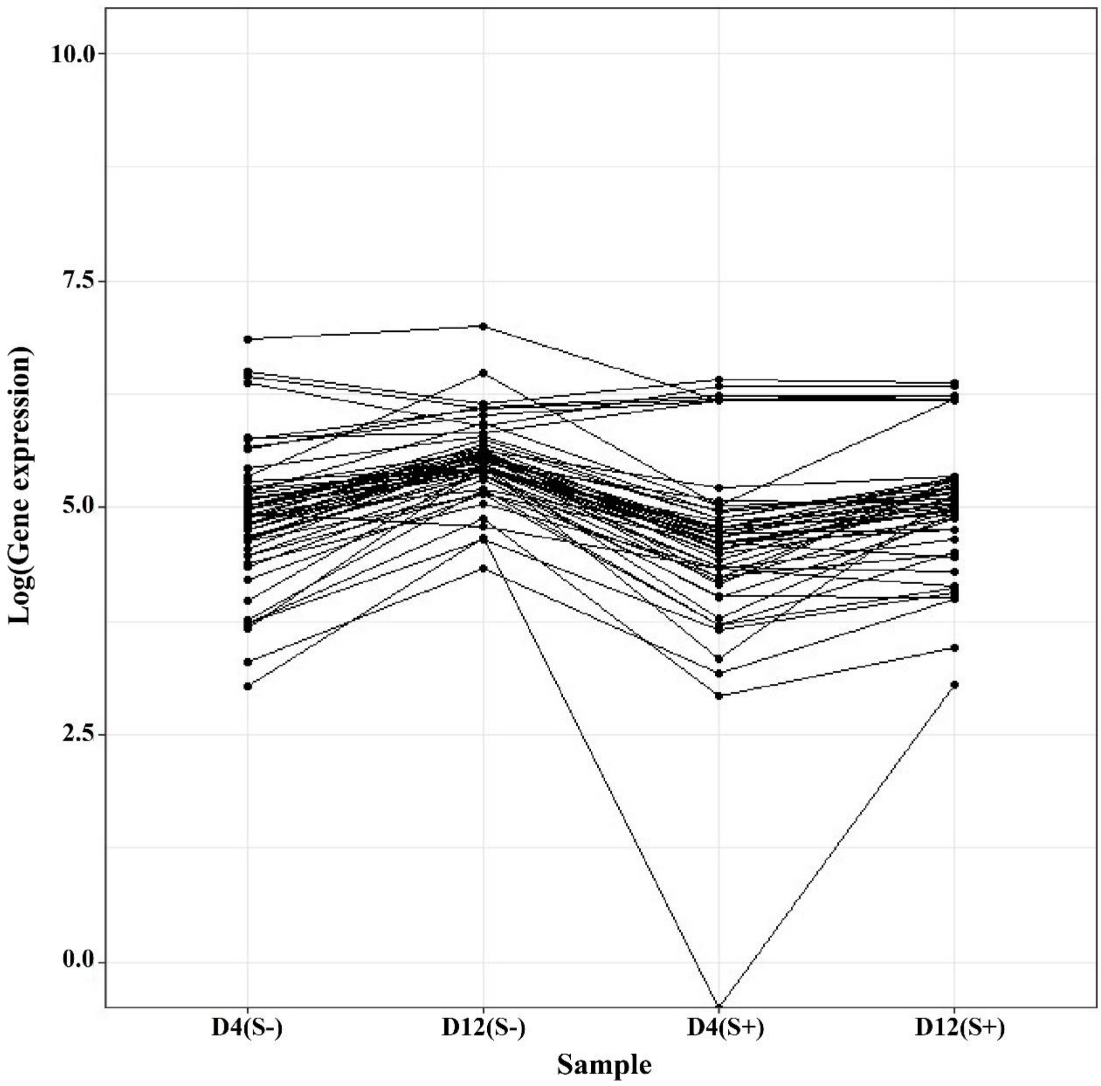

Supplementary Figure 14. The downregulated genes correlated with the motility in hypothetical chronology.

Supplementary Figure 15

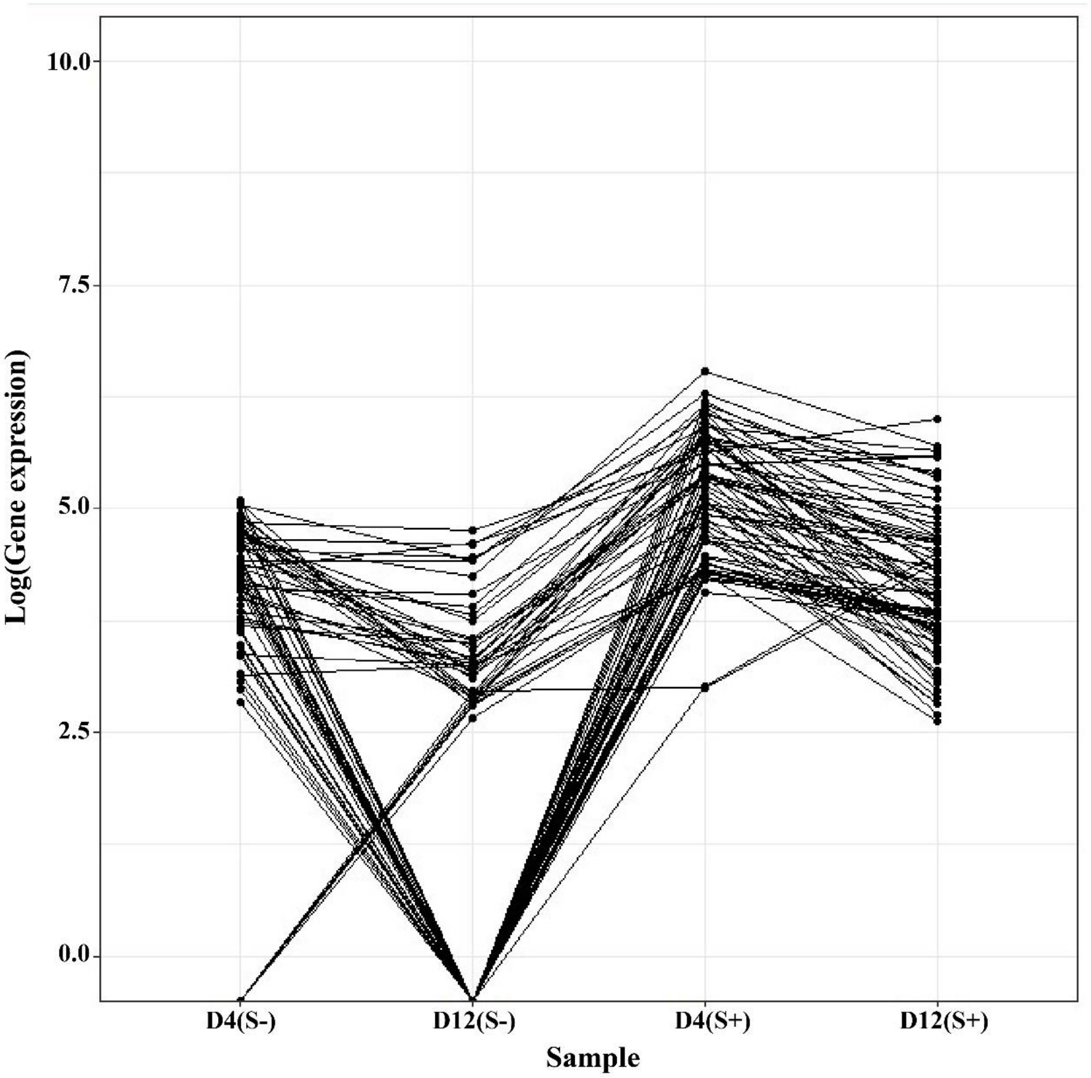

Supplementary Figure 15. The upregulated gene correlated with PT-diet in both the D4 and D12 samples, without distinguishing the day4 and day12 temporal difference.

Supplementary Figure 16

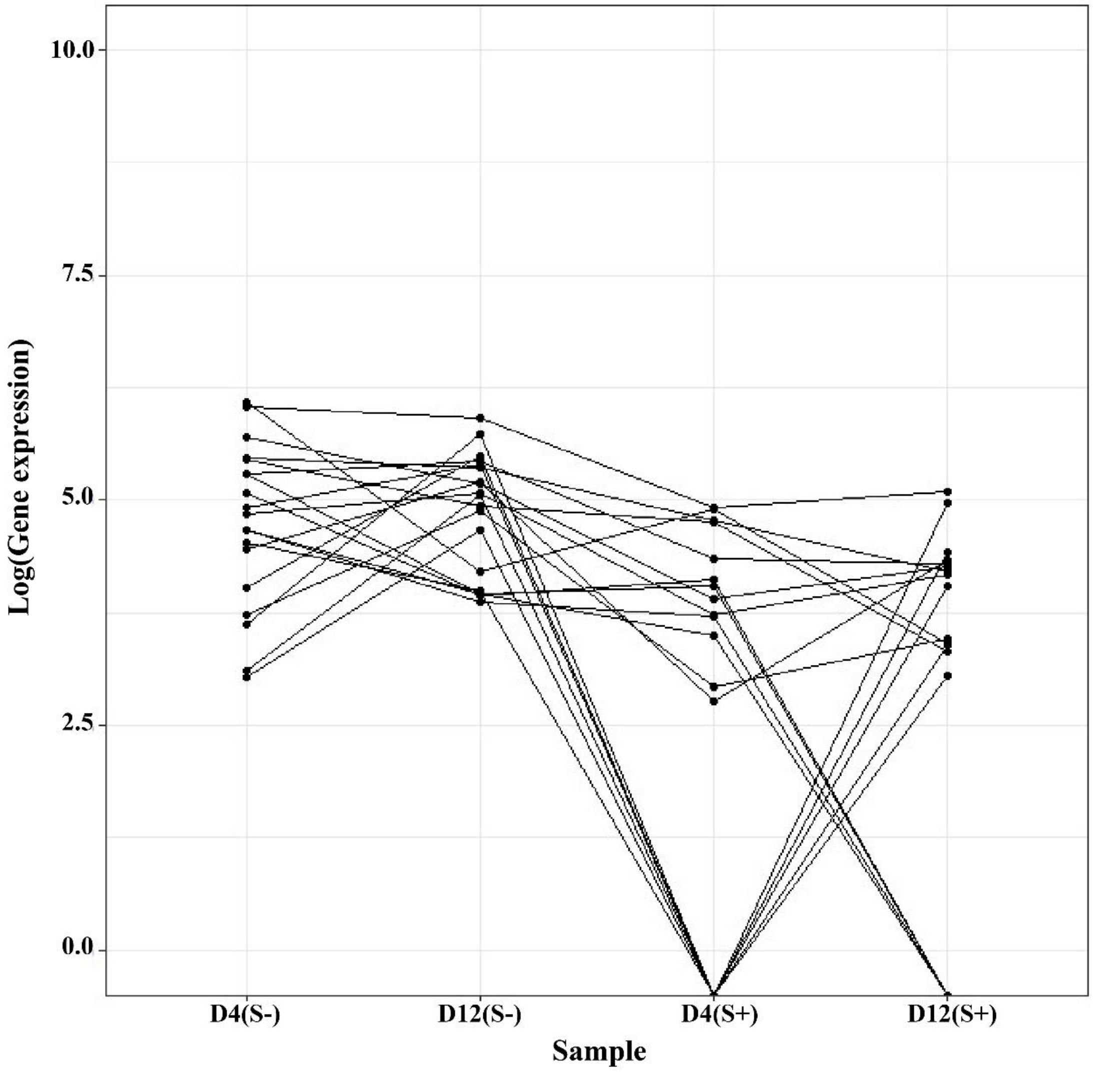

Supplementary Figure 16. The downregulated gene in both the D4 and D12 samples. They are ostf-4 / W02D7.9 / tbx-43 / Y116A8C.466 / fbxb-97 / dmsr-16 / C33D3.4 / ZK675.4 / R13H4.6 / F47B3.6 / dmsr-12 / Y105E8A.32 / clec-3 / col-135 / ins-20 / lys-10 / try-9.

Supplementary Figure 17

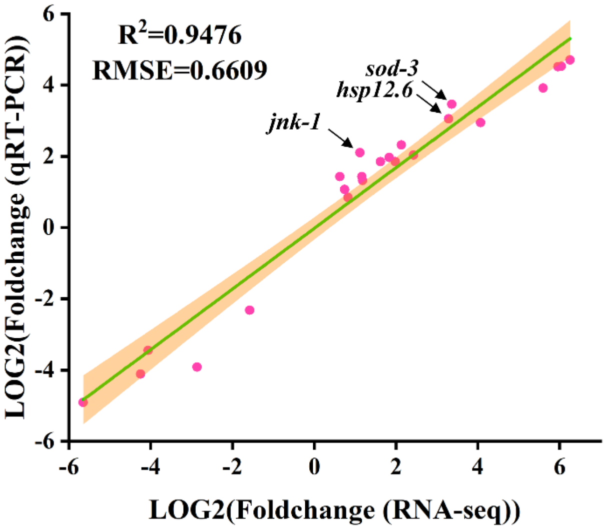

Supplementary Figure 17. Correlation between qRT-PCR and RNA-seq of *Caenorhabditis elegans* fed Dnd+ (S+) and Dnd- (S-) OP50.

Supplementary Figure 18

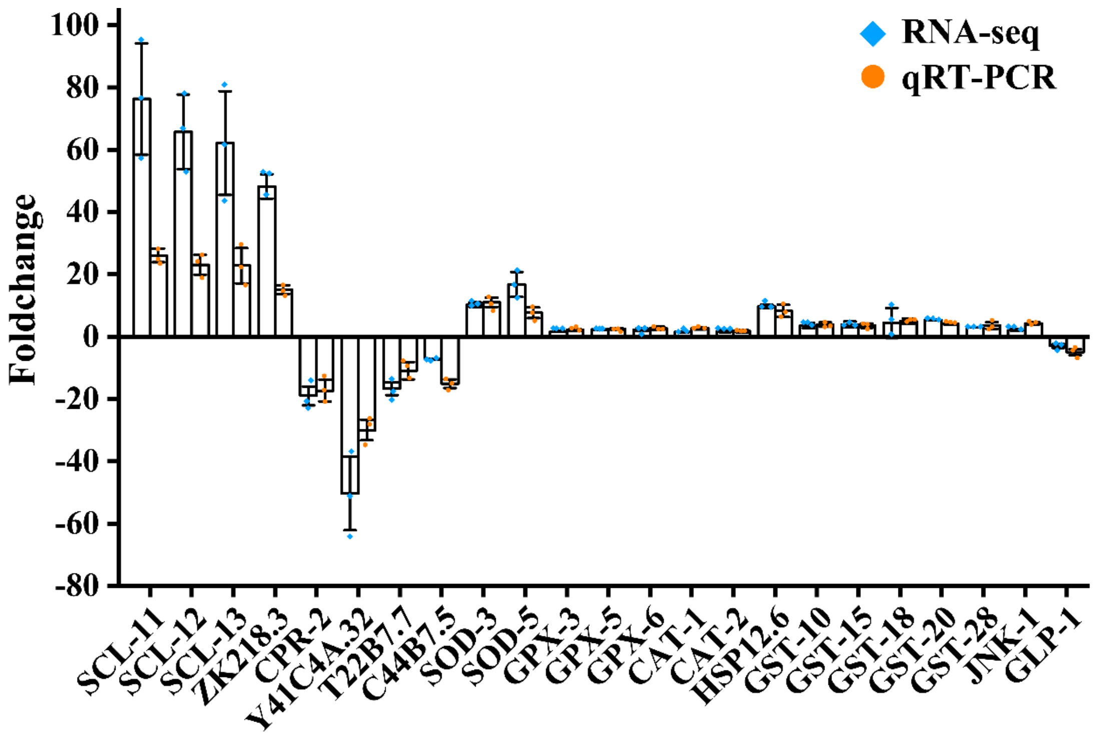

Supplementary Figure 18. qRT-PCR of different expression genes compared with RNA-seq. Error bars note the standard deviation.

Supplementary Figure 19

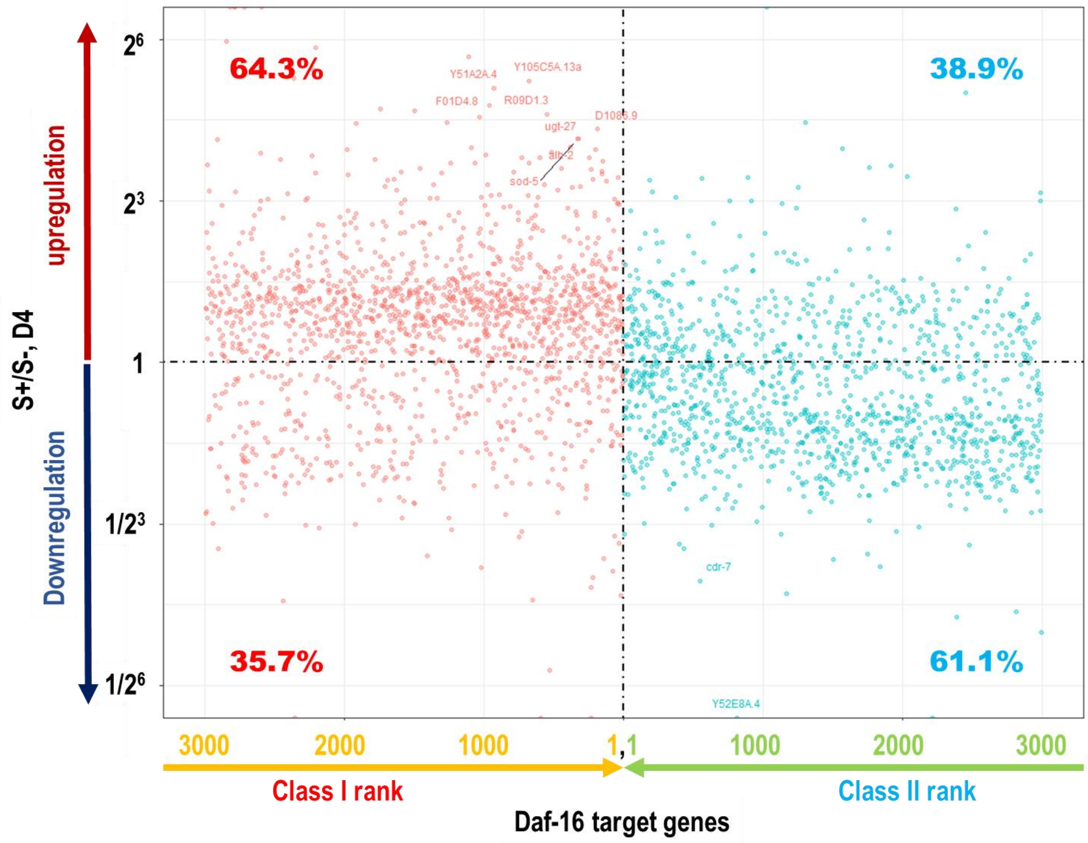

Supplementary Figure 19. Comparison of PT-diet gene regulation and *daf*-16 target genes from 46 contrasts of microarray data[1]. 8174 gene expressions of 19,286 *daf*-16 target were detected in this work, including 4178 of Class I and 3996 of Class II. PT-diet regulations exhibit the similar tendency as in *daf*-16, that is, 64.3% of the Class I genes are upregulated and 61.1% of the Class II genes downregulated. The highly correlated regulations are marked in the top-1000 genes, using a gene expression regulation threshold of $|\log_2(S+/S-)| > 4$ in the D4 samples.

Supplementary Figure 20

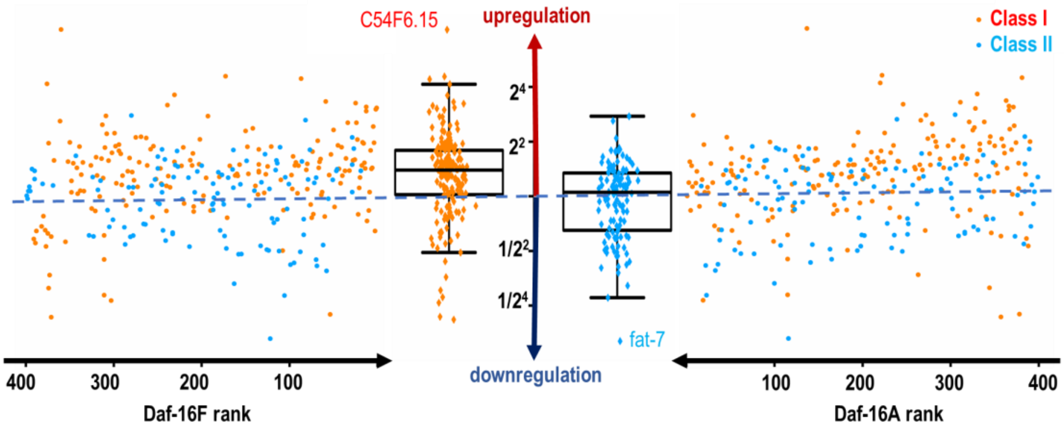

Supplementary Figure 20. Comparison with the genes specific in *daf*-16A and *daf*-16F isoforms [2]. Among 399 genes specified in *daf*-16A and *daf*-16F isoforms, 306 gene expression were detected in this work. Left and right, the 306 PT-diet regulated genes mapping to daf-16F and daf-16A Rank indexes, respectively; middle, the statistic box plots of daf-16-target Class I and Class II in the 306 genes, which are colored in orange and blue, respectively. The PT-diet regulation is different from either *daf*-16A or *daf*-16F isoform, though the *daf*-16-up genes are more significantly upregulated than the *daf*-16-down genes. In particular, *fat*-7 is dramatically downregulated by PT-diet.

Supplementary Figure 21

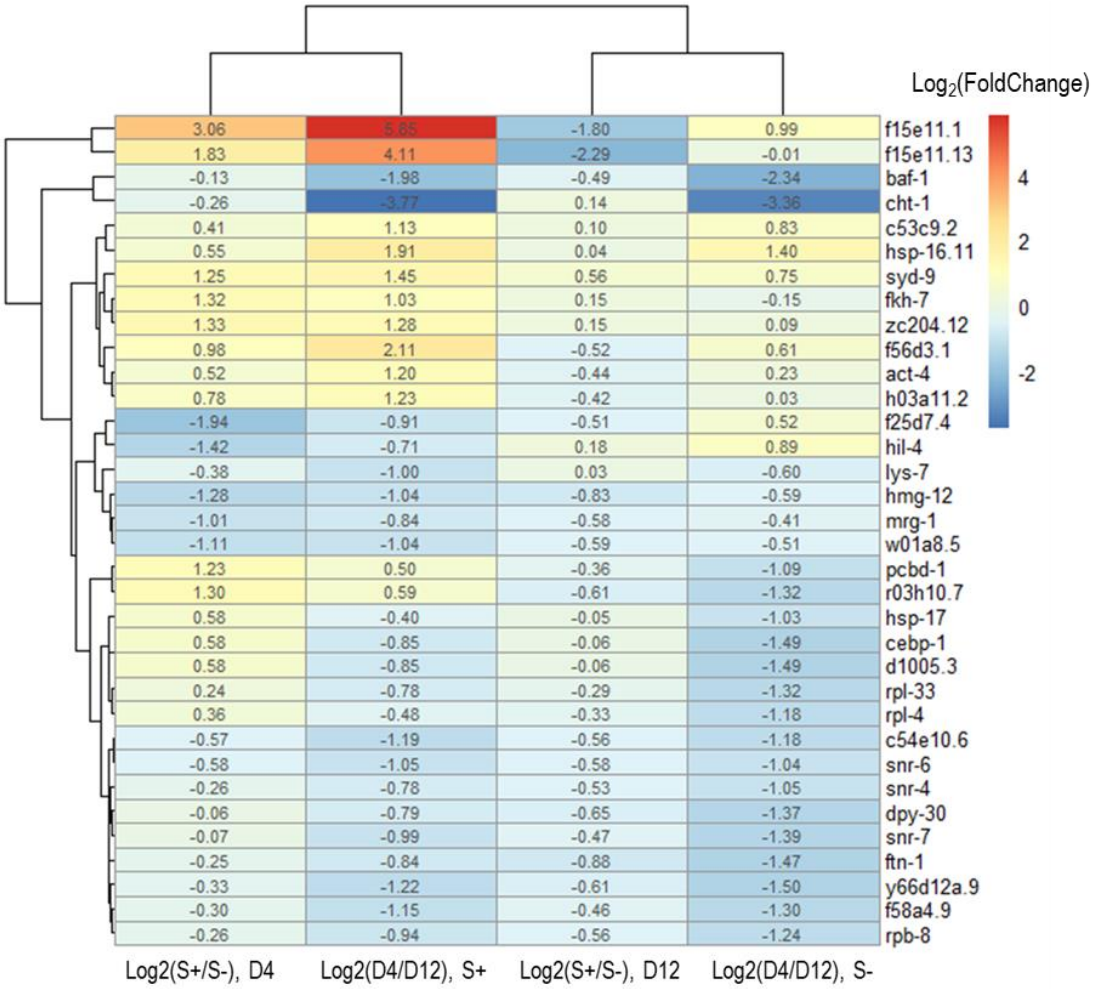

Supplementary Figure 21. Heatmap of the PT-diet regulation overlapped with daf-16 cofactors SWI/SNF-related genes [3]. PT-diet effect is considerably correlated with the aging from youth (D4) to senior (D12). F15E11.1/F15E11.13/*syn*-9/*fkh*-7/ZC204.12/*pcbd*-1/R03H10.7 were upregulated by PT-diet, while F25D7.4/*hil*-4/*hmg*-12/*mrg*-1/W01A8.5 were downregulated. Intriguingly, chromatin factor *baf*-1 was top-ranked in the PT-diet gene regulation.

Supplementary Table

Supplementary Table 1: Summary of relative fluorescence units of *C. elegans* (N2) fed on OP50 (S^-) and OP50 (S^+) at Day 1, 4, 8, and 12, after L4 larva-to-adult molts at 22 ºC.

DCF(row)	Mean(S+)	SE	Mean(S-)	SE	change (%)	p-value
D1	$9.5x10^4$	$2.7x10^4$	$1.2x10^5$	$2.5x10^4$	19.6	8.90E-05
D4	$1.2x10^5$	$3.5x10^4$	$1.5x10^5$	$3.7x10^4$	23.8	1.27E-05
D8	$1.3x10^5$	$5.1x10^4$	$1.9x10^5$	$4.3x10^4$	29.9	6.63E-07
D12	$2.4x10^5$	$1.0x10^5$	$2.2x10^5$	$9.7x10^4$	-6.7	0.2772
DCM(row)	Mean(S+)	SE	Mean(S-)	SE	change (%)	p-value
D1	$9.1x10^4$	$3.1x10^4$	$1.1x10^5$	$3.1x10^4$	20.7	0.0053
D4	$7.9x10^4$	$2.8x10^4$	$1.8x10^5$	$6.0x10^4$	56.7	7.94E-12
D8	$1.6x10^5$	$5.8x10^4$	$1.9x10^5$	$4.9x10^4$	18.2	0.0049
D12	$1.7x10^5$	$5.0x10^4$	$1.8x10^5$	$4.9x10^4$	4.8	0.4181
Lipofuscin (row)	Mean(S+)	SE	Mean(S-)	SE	change (%)	p-value
D1	$8.6x10^4$	$2.2x10^4$	$1.2x10^5$	$2.4x10^4$	25.4	7.05E-05
D4	$1.2x10^5$	$3.4x10^4$	$1.5x10^5$	$5.0x10^4$	23.2	4.65E-04
D8	$1.5x10^5$	$5.2x10^4$	$1.7x10^5$	$4.6x10^4$	12.3	0.1281
D12	$1.5x10^5$	$6.7x10^4$	$1.7x10^5$	$6.0x10^4$	9.1	0.3064

body size	D1	D4	D8	D12
S+	0.11±0.02	0.14±0.01	0.16±0.02	0.17±0.01
S-	0.11±0.01	0.15±0.01	0.15±0.02	0.16±0.01

DCF (normalized)	Mean(S+)	SE	Mean(S-)	SE	change (%)	p-value
D1	$7.6x10^5$	$2.2x10^5$	$9.4x10^5$	$2.0x10^5$	18.8	3.39E-05
D4	$8.3x10^5$	$2.5x10^5$	$1.0x10^6$	$2.5x10^5$	19.1	0.0005
D8	$8.4x10^5$	$3.2x10^5$	$1.3x10^6$	$2.8x10^5$	32.7	4.28E-08
D12	$1.5x10^6$	$6.4x10^5$	$1.5x10^6$	$6.6x10^5$	0.9	0.9306
DCM (normalized)	Mean(S+)	SE	Mean(S-)	SE	change (%)	p-value
D1	$7.3x10^5$	$2.5x10^5$	$9.1x10^5$	$2.5x10^5$	19.9	0.0075
D4	$5.6x10^5$	$2.0x10^5$	$1.2x10^6$	$4.0x10^5$	54	5.44E-11
D8	$9.8x10^5$	$3.7x10^5$	$1.2x10^6$	$3.2x10^5$	21.5	0.0005
D12	$1.1x10^6$	$3.1x10^5$	$1.2x10^6$	$3.3x10^5$	11.6	0.0459
Lipofuscin (normalized)	Mean(S+)	SE	Mean(S-)	SE	change (%)	p-value
D1	$6.9x10^5$	$1.8x10^5$	$9.2x10^5$	$1.9x10^5$	24.7	0.0001
D4	$8.4x10^5$	$2.5x10^5$	$1.0x10^6$	$3.4x10^5$	18.4	0.0055
D8	$9.4x10^5$	$3.3x10^5$	$1.1x10^6$	$3.0x10^5$	15.8	0.0476
D12	$9.7x10^5$	$4.2x10^5$	$1.1x10^6$	$4.0x10^5$	15.6	0.0691

Supplementary Table 2: Summary of lifespan of *C. elegans* (N2) fed on OP50 (S^-) and OP50 (S^+) and *daf-2* and *daf-16* fed on OP50.

Strains/Food	Mean lifespan [a] (days)	Percentage change [b]	Maximum lifespan [c] (days)	Number of worms	*p* value [d]
N2/OP50	16.6 ± 0.8	/	28.1 ± 0.7	132	/
N2/OP50 (S^+)	20.2 ± 1.4	21.7%	30.1 ± 0.6	124	0.0013
daf-2/OP50	30.3 ± 1.6	82.5%	37.2 ± 1.3	117	<0.001
daf-16/OP50	13.2 ± 0.6	-20.5%	23.6 ± 0.7	114	<0.001

[a] Mean lifespan = $\frac{1}{n}\sum_j \frac{x_j+x_{j+1}}{2} dj$, where j is the age category, dj is the number of worms that died in theage interval (x_j, x_{j+1}), and n is the number of uncensored worms.

[b] percentage change was calculated by (T − C)/C*100, where T is the mean survival time of worms fed on S+ strains and C is the mean survival time of worms fed on OP50

[c] The maximum lifespan is the time at which survival equals 0%

[d] The *p* value was calculated using the log-rank test by comparing the PT-fed group with the control

Supplementary Table 3 lifespans of *C. elegans* (N2) fed on alive and dead-OP50, OP50 (S-) and OP50 (S+).

C. elegans diet	Mean lifespan (days)	Change	Maximum lifespan (days)	Number of worms	*p* value*
OP50, alive	16.6 ± 0.8	/	27	124	/
OP50 (S^-), alive	17.1 ± 0.7	3.0%	28	120	0.65
OP50 (S^+), alive	20.2 ± 1.4	21.7%	31	132	<0.001
OP50, dead	18.8 ± 0.6	/	29	128	/
OP50 (S-), dead	19.4 ± 0.7	3.2%	29	119	0.30
OP50 (S+), dead	21.5 ± 1.5	14.3%	32	127	<0.001

* The statistical significance was calculated with log-rank test.

Supplementary Table 4 lifespans of *C. elegans* (*daf-2* and *daf-16*) fed on alive and dead-OP50, OP50 (S-) and OP50 (S+)

C. elegans and diet	Mean lifespan (days)	Change	Maximum lifespan (days)	Number of worms	*p* value
daf-2 (long-lived)					
OP50, alive	30.3 ± 1.6	/	40.2 ± 1.3	117	/
OP50 (S^-), alive	30.8 ± 1.2	1.7%	40.6 ± 1.4	114	0.3
OP50 (S^+),alive	31.8 ± 1.2	5.0%	43.6 ± 1.7	117	0.01
OP50,dead	29.2 ± 1.8	/	40.4 ± 0.9	123	/
OP50 (S-), dead	29.7 ± 1.0	1.6%	40.8 ± 1.2	116	0.98
OP50 (S+),dead	30.6 ± 1.6	4.3%	42.6 ± 1.7	121	0.02
daf-16(short-lived)					
OP50, alive	13.2 ± 0.6	/	23.6 ± 0.7	89	/
OP50 (S-), alive	13.5 ± 0.9	2.3%	23.9 ± 0.8	85	0.86
OP50 (S^+), alive	15.0 ± 1.3	13.6%	25.8 ± 0.9	89	0.03
OP50, dead	15.3 ± 0.7	/	26.1 ± 0.7	83	/
OP50 (S-), dead	15.5 ± 1.0	1.3%	25.9 ± 1.1	86	0.64
OP50 (S+), dead	17.3 ± 1.1	13.0%	27.7 ± 1.4	83	0.003

Supplementary Table 5: The lifespan of *C. elegans* (N2) under the stress of paraquat, Cr^{6+}, heat (35 °C) and UV.

Strains/Food	Mean lifespan [a] (hours)	Percentage change [b]	Maximum lifespan [c] (hours)	Number of worms	*p* value [d]
Paraquat					
N2/OP50	83.2 ± 20.0		132	138	
N2/OP50 (S^+)	93.3 ± 31.8	12.14	168	146	0.02
Cr^{6+}					
N2/OP50	60.9 ± 21.8		108	111	
N2/OP50 (S^+)	71.5 ± 31.6	17.41	144	110	0.01
Heat (35 °C)					
N2/OP50	13.8 ± 6.8		28	146	
N2/OP50 (S^+)	16.2 ± 7.3	17.39	32	215	0.004
UV					
N2/OP50	179.7 ± 41.9		240	41	
N2/OP50 (S^+)	152.0 ± 42.6	-15.56	240	38	0.001

[a] Mean lifespan = $\frac{1}{n}\sum_j \frac{x_j+x_{j+1}}{2} dj$, where j is the age category, dj is the number of worms that died in the age interval (x_j, x_{j+1}), and n is the number of uncensored worms.

[b] percentage change was calculated by (T − C)/C*100, where T is the mean survival time of worms fed on OP50 (S+) and C is the mean survival time of worms fed on OP50

[c] The maximum lifespan is the time at which survival equals 0%

[d] The *p* value was calculated using the log-rank test by comparing the PT-fed group with the control

Supplementary Table 6: Effects of DETP on the lifespan of *C. elegans* under stress induced by paraquat and Cr^{6+}.

**paraquat**	**mean lifespan**	**change (%)**	**Maximum lifespan**	**NO. of worms**	**p-value**
control	81.1 ± 28.6		132	260	
0.6 mM DETP	84.3 ± 34.5	3.95	168	267	0.06
5.0 mM DETP	100.3 ± 39.6	23.67	192	225	p<0.001
10.0 mM DETP	110.4 ± 46.2	36.13	216	213	p<0.001
**Cr^{6+}**					
control	58.5 ± 15.9		120	253	
0.6 mM DETP	55.5 ± 16.2	-5.12821	132	265	0.17
5.0 mM DETP	65.3 ± 23.0	11.62393	144	223	0.0073
10.0 mM DETP	57.8 ± 14.7	-1.19658	120	241	0.63

Supplementary Table 7: The number of different express genes in *C. elegans* (N2) fed on OP50 (S^-) and OP50 (S^+).

**compare**	**all**	**up**	**down**	**threshold**
POS_4dvsNEG_4d	4662	2271	2891	DESeq2 padj<0.01 |log2FoldChange|>1.0
POS_12dvsPOS_4d	6314	3281	3033	DESeq2 padj<0.01 |log2FoldChange|>1.0
POS_12dvsNEG_12d	2311	1931	380	DESeq2 padj<0.01 |log2FoldChange|>1.0
NEG_12dvsNEG_4d	4678	1804	2874	DESeq2 padj<0.01 |log2FoldChange|>1.0
POS_12dvsNEG_4d	1752	555	1836	DESeq2 padj<0.01 |log2FoldChange|>1.0

Supplementary Table 8. Top-100 upregulated genes correlated with the temporality of ROS-response, in the decreasing order of gene-expressions in D4(+) samples. (highlighted: plausible ROS-related)

Genes (1-25)	Genes (26-50)	Genes (51-75)	Genes (76-100)
C41G6.13	K02G10.5	F45D11.1	ZK218.11
F53F4.7	Y60A3A.23	F48G7.8	ZC376.8
impt-1	daf-12	C06H5.12	K09D9.9
H14N18.2	F01D5.7	Y55F3AM.14	ssu-1
C14A4.9	B0238.7	linc-62	T05D4.3
Y4C6B.4	F15H10.5	F45D11.15	C44B11.1
phat-5	C03G6.6	F07C4.12	C40C9.4
smf-2	F32G8.2	oac-41	EGAP9.4
Y51A2A.12	clec-174	K09E2.1	scav-4
T05A8.6	Y38E10A.9	C46A5.4	cutl-28
pola-1	B0478.3	dsl-7	C13A2.5
nspb-1	T23F1.5	F15H10.10	R05G9R.1
R13D11.11	Y52B11B.1	BE0003N10.3	unc-79
T23F6.5	grl-21	bus-17	M153.2
sqt-1	C16C4.17	lad-2	F32D8.1
grl-25	C33B4.5	F54C8.1	F53F4.15
grd-4	C32H11.3	sek-5	C45H4.13
ent-7	mltn-7	lipl-6	linc-61
Y71H2AR.2	clec-20	prmt-4	F30A10.12
F56H9.8	F32D8.2	clec-21	C08A9.3
srj-32	T01D1.8	mam-2	syd-1
C13A2.1	F38C2.1	srh-146	F35F10.19
K02C4.2	ZK822.9	W06G6.16	unc-3
gpr-2	novel.60	C13A2.2	T19C9.10
ttr-52	ptr-10	C02F4.4	F23H11.6

Supplementary Table 9 Top-100 downregulated genes correlated with the temporality of ROS-response, in the increasing order of gene-expressions in D4(+) samples. (highlighted: plausible ROS-related).

Genes (1-25)	Genes (26-50)	Genes (51-75)	Genes (76-100)
novel.123	T20B12.4	fbxb-15	str-41
novel.76	F10A3.1	F40G12.7	M151.3
str-45	ZK262.8	C17E4.2	novel.8
F19H6.3	nhr-74	F15A4.2	srx-13
scl-19	fbxa-200	K01G12.3	C31G12.1
ZK678.3	srx-14	Y49F6A.5	F58D2.4
F53G2.8	W04E12.2	fkh-5	cutl-13
K02F6.6	asp-18	F53G2.2	btb-7
W02D7.9	nlp-39	W06D11.2	F33E2.4
plep-1	E03A3.5	R13H4.6	novel.52
Y81B9A.2	F52C6.14	Y37H2A.13	T11F8.5
ZC239.20	K11H12.3	cyp-13B2	C49F5.5
tbx-43	C47F8.1	fbxc-28	novel.111
B0513.6	fbxc-42	his-67	C08F11.12
fbxb-97	ins-34	W03D8.7	F54F7.9
clec-176	B0281.4	Y80D3A.11	R155.4
srbc-12	set-13	linc-116	bath-23
fbxb-54	F31F6.1	F08F1.9	cdr-7
dmsr-16	scl-27	R09A8.1	novel.5
Y47H10A.2	F22F4.5	his-66	ceh-86
gadr-4	R160.5	scl-9	W04G3.13
Y73B3A.7	F22H10.1	W04A8.5	fbxa-181
scl-24	Y105C5B.20	T22C8.4	Y37H2A.10
T10D4.1	fbxa-194	E02H9.1	clec-97

Supplementary Table 10. Top-100 upregulated genes correlated with the temporality of motility, in the decreasing order of the D12(+) values.

Genes (1-25)	Genes (26-50)	Genes (51-75)	Genes (76-100)
gst-24	F47D12.3	F15H10.10	C13A2.1
T28H11.7	ent-7	asp-9	clec-24
ZK945.7	phat-5	C32H11.4	Y51A2A.12
F13A7.1	fbxa-164	Y47D3B.1	F15H10.6
C30G7.3	hot-5	Y46H3C.7	D2092.8
F32B6.4	tag-96	C42D4.3	clec-33
R09E10.2	C16C4.17	T05B4.12	glf-1
E03H12.7	Y95B8A.2	grl-25	K09D9.9
T14G10.8	C45E5.4	F36D1.15	C13A2.2
Y47G6A.26	noah-2	grl-21	nspb-8
tbx-34	elt-1	C02E7.7	F29D10.2
F56B3.6	E02H4.4	col-14	srx-19
F53G12.8	ztf-14	ugt-53	lpr-5
R02D5.7	C24H10.3	ceh-8	ZK105.12
F58D5.7	F10B5.3	C52E2.2	C43F9.11
R07E5.15	tyr-3	srj-32	F15H10.5
W01B6.2	D2096.10	C56C10.4	Y105C5A.1271
spe-46	paqr-3	C16C4.1	chil-19
sst-20	nhr-25	F53F4.15	F09E10.6
T02E1.6	F19C6.5	asm-3	oac-12
C09F9.1	Y4C6B.4	C02E7.6	F59B2.11
C43E11.5	sulp-5	Y53G8AM.5	ZK1055.4
ssp-19	Y9D1A.1	tag-297	clec-32
C06H5.11	C16D9.1	dpy-7	nspb-9
impt-1	K10H10.5	srd-45	F55C9.12

Supplementary Table 11. Downregulated genes correlated with the temporality of motility, in the increasing order of gene-expressions in D12(+) samples.

Genes (1-15)	Genes (15-30)	Genes (31-45)	Genes (46-53)
dmsr-16	cutl-13	ZK380.5	B0041.11
clec-161	C24D10.5	fbxa-94	K09H11.11
his-43	Y55D5A.2	M01G12.14	K02D10.8
T25D1.2	mct-2	T08D2.5	F55D12.5
C11G6.2	Y74C9A.1	math-49	Y69H2.9
nhr-181	Y17G7B.23	F11A5.18	nhr-23
clec-3	ceh-99	set-28	gld-2
F07C6.6	K09E3.6	Y53G8AL.1	
novel.66	M04C9.4	bath-34	
K01G12.3	nhx-6	srw-33	
npp-26	his-12	arrd-9	
fbxa-84	Y51A2D.8	cyp-33E3	
F14B6.4	nhr-74	fbxa-7	
F14H3.3	C02E7.10	Y17D7B.7	
ZK892.6	srsx-25	eat-4	

Supplementary Table 12. Upregulated genes in both the D4 and D12 samples, with the threshold value of 2^2.

Genes (1-20)	Genes (21-40)	Genes (41-60)	Genes (61-78)
C41G6.13	pqn-98	clec-249	F45D11.2
Y51A2A.12	C06H5.13	novel.38	srx-35
T05A8.6	F55C9.6	C09F9.4	str-51
R13D11.11	C16C4.1	Y97E10B.1	srw-124
Y60A3A.23	F36D1.15	F43C11.5	srd-68
B0238.7	T28A11.3	clec-248	C55A1.13
F15H10.5	C13C4.8	grl-29	C06C6.1
eak-4	ZK1025.3	lgc-36	srh-138
C33B4.5	grd-8	chil-19	srh-147
mltn-7	F20C5.7	F15H10.6	Y9C9A.17
clec-20	F36D1.5	srx-113	srw-139
F45D11.1	Y53G8AM.5	T23D5.5	ZK1025.4
linc-62	T05B4.10	srx-128	srw-130
F07C4.12	F21C10.5	F54D12.7	T05H4.3
oac-41	F55C9.12	srbc-21	Y19D10B.8
F15H10.10	novel.79	sru-31	srx-52
C17B7.15	C52E2.2	T03D3.13	
clec-21	F36D1.10	srx-19	
W06G6.16	C31B8.2	str-58	
C13A2.2	srx-95	srh-245	

Supplementary Table 13. Top-100 upregulated genes in the D4 samples. (red, ROS-related; purple, ROS+longevity)

Genes (1-25)	Genes (26-50)	Genes (51-75)	Genes (76-100)
scl-12	F47B8.18	C29F5.8	F14D7.5
scl-13	Y119D3B.21	T04C12.7	ugt-27
ZK218.3	C54F6.17	col-72	Y60C6A.2
scl-11	hsp-12.6	ZK218.11	T23F2.4
ZK218.1	lys-3	col-108	R08F11.7
spp-11	cyp-34A2	F48G7.7	F20A1.6
F57F4.2	ttr-19	dur-1	C16C4.17
col-51	tyr-3	lips-15	nhr-246
fil-1	C08E3.13	grd-3	ttr-26
Y59A8B.26	F01D4.8	clec-12	W04E12.7
col-183	C18H7.1	cyp-13B1	cyp-29A3
Y59A8B.19	col-2	fbxb-98	kvs-5
R05A10.1	F47B8.14	grd-4	col-84
F46F2.3	Y26G10.7	grl-23	grd-10
oac-39	T05B4.12	C08E3.1	Y39H10A.1
sod-3	F23F1.2	spi-1	F41E7.20
F47B8.13	asp-16	T14A8.2	col-43
col-85	C54F6.15	alh-2	F08H9.4
T26C12.2	F16B4.5	spp-20	cyp-13A2
T22F3.11	col-37	C07F11.2	R11D1.4
Y54G2A.57	D1086.3	K08D12.7	ZK218.7
col-149	dmd-10	T04C12.1	nas-10
col-40	F10B5.3	gst-20	cut-1
ZK218.5	sod-5	C23G10.11	ZK355.3
F10C2.7	col-150	Y50E8A.19	fipr-25

Supplementary Table 14. Top-100 downregulated genes in the D4 samples. (red, ROS-related; green, longevity-related; purple, ROS+longevity)

Genes (1-25)	Genes (26-50)	Genes (51-75)	Genes (76-100)
cpr-2	gipc-2	pos-1	scl-20
T22B7.7	msrp-2	gyg-2	ddo-3
Y41C4A.32	pmt-2	Y75B12B.1	gln-5
C44B7.5	C33F10.1	C39D10.7	gfat-2
C30G12.2	sss-1	nspd-6	C14C10.1
lys-4	F18A1.7	arf-1.1	tbh-1
perm-4	mesp-1	F27C1.1	C04F12.7
acdh-1	sams-1	msp-45	egg-5
F48E3.4	cpg-1	nspd-9	mex-1
B0513.4	T01C3.3	K06A5.2	C02F5.5
C10G8.4	T23B3.5	mex-6	msp-59
metr-1	C10G11.8	cpg-2	Y69E1A.1
T04G9.7	puf-5	T22D1.5	ZK550.5
mmaa-1	F36A2.10	egg-1	egg-4
T05E12.6	sdz-27	memi-3	rme-2
perm-2	clec-88	puf-3	pudl-1
dhs-26	lips-6	EEED8.3	Y106G6G.4
ZK813.2	C15C6.2	cbd-1	gln-6
pmp-5	nspd-2	C05B5.2	inx-8
cyb-2.2	Y59E9AL.6	clec-87	R09E10.2
R09E10.6	asp-13	ZK813.1	T12G3.6
vit-2	C17G1.2	clec-47	msp-142
trcs-1	W06B4.1	puf-7	ZK858.2
F08B4.8	F07A5.2	gln-2	gipc-1
T13F2.9	ZK938.1	nos-2	C01G8.1

Supplementary Table 15. Regulatory profiles of target genes related to DAF-16 / HSF-1 / SKN-1. (FPKM values of the significantly regulated genes) [4-7]

Genes	D4(S-)	D12(S-)	D4(S+)	D12(S+)
cep-1	14.2	13.1	7.0	11.9
gbb-1	4.2	3.3	8.4	4.1
pal-1	32.2	24.5	10.3	17.7
vrk-1	23.9	23.3	11.7	19.5

Supplementary Table 16: Stress response genes of *C. elegans* were induced by PT

Name	Foldchange[#] (D4)	padj (D4)	Foldchange (D12)	padj (D12)
sod-3	10.25	8.73E-110	/	/
sod-5	16.70	1.42E-64	3.04	9.74E-08
gpx-5	2.27	2.40E-19	/	/
gpx-6	2.25	2.86E-07	/	/
hsp12.6	9.73	1.50E-89	5.27	8.47E-18

[#]Foldchange represents the FPKM ratio between *C. elegans* (S+) and *C. elegans* (S-)

Supplementary Table 17. Aging-related genes that are regulated by PT-diet

Genes	D4(S-)	D12(S-)	D4(S+)	D12(S+)	Aging
**insulin/ IGF-1 signaling pathway**					
mtl-1	2077	5956	7112	6672	*anti-*
col-10	1943	120	4561	424	*anti-*
col-13	1905	66	2613	6	*anti-*
daf-7	347	479	833	703	*pro-*
daf-12	1905	1207	6456	1724	*anti-*
daf-18	4663	1777	1268	2214	*anti-*
daf-28	214	172	505	267	*pro-*
jnk-1	189	162	410	199	*anti-*
mes-4	1324	792	497	860	*anti-*
pgl-1	5217	1735	2257	2368	*Unknown*
pgl-3	2176	1240	712	1408	*Unknown*
pie-1	760	274	234	247	*anti*
**Dietary restriction pathway**					
pha-4	761	596	1557	828	*anti-*
ins-7	343	177	91	708	*anti-*
sams-1	9579	1146	2354	1114	*anti-*
**TOR pathway**					
pha-4	761	596	1557	828	*anti-*
**SN pathway**					
ttx-1	184	254	402	235	*anti-*
ser-3	160	170	475	181	*pro-*
**RPD pathway**					
glp-1	2283	947	768	891	*anti-*
fat-6	26096	8088	12465	8745	*Unknown*
lips-17	422	463	564	168	*Unknown*
**MIT pathway**					
sod-3	237	299	2436	409	*anti-*
ced-3	1831	888	439	925	*Unknown*

*PT-diet anti-aging and pro-aging are determined based on aging-resistance and aging promoting characterizations in literature. Upregulation of aging-resistance gene indicates anti-aging, and *vice versa*. (the gray highlight notes gene regulations opposite to the observed phenotype) [8-24]

Supplementary Table 18. comparation of different expression genes by qRT-PCR and RNA-seq （qRT-PCR is based on PMP-3 as the internal reference gene）

Gene ID	Gene Name	Foldchange (RNA-seq)	Foldchange (qPCR) ± SEM
WBGene00009892	SCL-11	76.3	26.1 ± 2.2
WBGene00019178	SCL-12	65.8	23.1 ± 3.2
WBGene00019179	SCL-13	62.2	22.8 ± 5.6
WBGene00013937	ZK218.3	48.3	15.1 ± 1.4
WBGene00000782	CPR-2	1/19.0	1/17.3 ± 3.5
WBGene00270321	Y41C4A.32	1/50.3	1/30.0 ± 3.3
WBGene00020674	T22B7.7	1/16.7	1/10.9 ± 2.8
WBGene00016627	C44B7.5	1/7.3	1/15.1 ± 1.4
WBGene00004932	SOD-3	10.3	11.0 ± 1.5
WBGene00007036	SOD-5	16.8	7.7 ± 1.7
WBGene00007517	GPX-3	1.7	2.1 ± 0.3
WBGene00007516	GPX-5	2.3	2.5 ± 0.3
WBGene00020373	GPX-6	2.2	2.7 ± 0.6
WBGene00000295	CAT-1	1.5	2.7 ± 0.4
WBGene00000296	CAT-2	1.8	1.8 ± 0.4
WBGene00002013	HSP12.6	9.7	8.3 ± 2.0
WBGene00001758	GST-10	3.6	3.9 ± 0.7
WBGene00001763	GST-15	3.9	3.6 ± 0.7
WBGene00001766	GST-18	4.4	5.0 ± 0.9
WBGene00001768	GST-20	5.4	4.1 ± 0.3
WBGene00001776	GST-28	3.1	3.6 ± 1.1
WBGene00002178	JNK-1	2.2	4.3 ± 0.5
WBGene00001609	GLP-1	1/3.0	1/5.0 ± 1.0

Supplementary Table 19. The top-ranking GO terms in the GO enrichment of *Caenorhabditis elegans fed* PT-containing bacterial food, long-term. (using day12 worms. ND: non-detected significant change)

GO ID	*Description*	*up*	*down*	p_{adj}
Down-regulation				
Cell component				
GO:0005581	collagen trimer	5	28	9.7×10^{-4}
GO:0000786	nucleosome	6	13	1.1×10^{-3}
GO:0044815	DNA packaging complex	6	13	2.1×10^{-3}
Biological process	*ND*			
Molecular function				
GO:0042302	structural constituent of cuticle	6	27	6.2×10^{-5}
Up-regulation				
Cell component				
GO:0031143	pseudopodium	36	0	2.3×10^{-29}
GO:0031234	extrinsic component	31	0	3.0×10^{-13}
GO:0009898	cytoplasmic side	33	0	3.7×10^{-12}
Biological process				
GO:0018107	peptidyl-threonine phosphorylation	76	1	8.7×10^{-54}
GO:0018210	peptidyl-threonine modification	76	1	6.8×10^{-53}
GO:0006470	protein dephosphorylation	99	0	7.4×10^{-53}
Molecular function				
GO:0004721	phosphoprotein phosphatase activity	95	0	8.2×10^{-51}
GO:0016791	phosphatase activity	99	3	2.5×10^{-45}
GO:0004672	protein kinase activity	133	2	1.6×10^{-39}

Supplementary Table 20. The qRT-PCR primers used in this work.

Gene	Forward (5'-3')	Reverse (5'-3')
PMP-3	CGGTGTTAAAACTCACTGGAGA	TCGTGAAGTTCCATAACACGA
SCL-11	GCCCAACTGGACACTCTCAG	TTCCCAGGATGAAGATGCGG
SCL-12	TGCTGGAACTATCGAGCCAC	TGTCAAGACTGGTTGGAGCC
SCL-13	CAAAGTTGCAGTGGTCTGCC	AAGCACAGAGCCCAGAATCC
ZK218.3	ATCACCACAGATGAGCCAGC	CTGCGCTTTGGCAACAGAAT
CPR-2	CGCTGTCAACTCTTGGGGAT	TATCTCGGCAATCCAGCGAC
Y41C4A.32	ATGCAAATTGCCGTGAACCC	ACCCTCCAGAGTGCAAATCG
T22B7.7	CAACGACAATTGCACAGCCA	TGCAGACAAAAGCGCTGAAC
C44B7.5	GATGTGTTGGAACCGAGTGC	AACCAATTGCAAGCCACGTC
SOD-3	CCAACCAGCGCTGAAATTCAATGG	GGAACCGAAGTCGCGCTTAATAGT
SOD-5	GAACTGCTGTCTTCGGAACTG	CCATGAAGTCCTGGTGACAAT
GPX-3	AAGAGCCAGCAGAGAACCAC	ATATCGGATGGCCGAACAGG
GPX-5	CGACTCGCTGGAGTCAATGT	ATGAGAAGCACCTGTCCACG
GPX-6	ATTTCGAGTTGCCGCGTTTC	GCTGATAATGATGAGCCACGC
CAT-1	CACGCTCTCCTCGGATTCTC	GGTCCGGAGAAGAATGGTCC
CAT-2	ATTGGTGCTGGGCTACTGTC	GCAACAAAGTAGAGCGGCTG
HSP12.6	TCCAGTGATGGCTGACGAAG	GGGAGGAAGTTATGGGCTTCT
GST-10	CGAAGACATTCGGTTCGACT	CGTTGGATCCGTTCAAGCCA
GST-15	CCATTTGGTCAGCTCCCAGT	TCTTCTTCTGGTTTCCCGGC
GST-18	ATTGGGGATGGCATCACGTT	CACTTTCTTTTGCAGAGCTACCA
GST-20	CGGACGAGGATTGGGAGATG	TTTGGAGTCCCGAACTGAGC
GST-28	ACCTTCGCGGACATTGTCAT	AGCCTCTACCCACTTCCTGA
JNK-1	CCGTCACATCCAGGTAGAAGC	GAACCAGCCAATTCCCAACG
GLP-1	TTCAACAGCGCAAAGTGTCG	TGCGAGACCATCCTTCATCG

Supplementary References

[1] Tepper R. G., Ashraf J., Kaletsky R., Kleemann G., Murphy C. T., & Bussemaker H. J. PQM-1 complements DAF-16 as a key transcriptional regulator of *daf*-2-mediated development and longevity. *Cell* **154**, 676-690 (2013).

[2] Chen A. T., Guo C., Itani O.A., Budaitis B.G., Williams T.W., Hopkins C. E., McEachin R. C., Pande M., Grant A.R., Yoshina S., Mitani S., & Hu P.J. Longevity genes revealed by integrative analysis of isoform-specific *daf*-16/FoxO mutants of *Caenorhabditis elegans*. *Genetics* **201**, 613-629 (2015).

[3] Riedel C.G., Dowen R.H., Lourenco G.F., Kirienko N.V., Heimbucher T., West J.A., Bowman S.K., Kingston R.E., Dillin A., Asara J.M., & Ruvkun G. DAF-16 employs the chromatin remodeller SWI/SNF to promote stress resistance and longevity. *Nature Cell Biology* **15**,491-501 (2013).

[4] Arum O. & Johnson T.E. Reduced expression of the *Caenorhabditis elegans* p53 ortholog cep-1 results in increased longevity. *J Gerontol A Biol Sci Med Sci*. **62**, 951-9 (2007).

[5] Schultheis C., Brauner M., Liewald J.F. & Gottschalk A. Optogenetic analysis of GABAB receptor signaling in *Caenorhabditis elegans* motor neurons. *J Neurophysiol*. **106**, 817-827 (2011).

[6] Chun L., Gong J., Yuan F., Zhang B., Liu H., Zheng T., Yu T., Xu X.Z., & Liu J. Metabotropic GABA signalling modulates longevity in *C. elegans*. *Nat Commun*. **6**, 8828 (2015).

[7] Park S., Artan M., Han S.H., Park H.H., Jung Y., Hwang A.B., Shin W.S., Kim K.T., & Lee S.V. VRK-1 extends life span by activation of AMPK via phosphorylation. *Sci Adv*.; **6**, eaaw7824 (2020).

[8] Lee, Y.J. et al. Genes and pathways that influence longevity in *Caenorhabditis elegans*. Aging Mechanisms. Springer, Tokyo, 123-169. (2015).

[9] Baesler J., Michaelis V., Stiboller M., Haase H., Aschner M., Schwerdtle T., Sturzenbaum S.R., & Bornhorst J. Nutritive Manganese and Zinc Overdosing in Aging *C. elegans* Result in a Metallothionein-Mediated Alteration in Metal Homeostasis. *Mol Nutr Food Res*. **65**, e2001176 (2021).

[10] Ewald C.Y., Landis J.N., Porter Abate J., Murphy C.T., & Blackwell T.K. Dauer-independent insulin/IGF-1-signalling implicates collagen remodelling in longevity. *Nature*. **519**, 97-101 (2015).

[11] Shaw W.M., Luo S., Landis J., Ashraf J., & Murphy C.T. The *C. elegans* TGF-beta Dauer pathway regulates longevity via insulin signaling. *Curr Biol*. **17**, 1635-45 (2007).

[12] Lee S.J., & Kenyon C. Regulation of the longevity response to temperature by thermosensory neurons in Caenorhabditis elegans. Curr Biol. **19**, 715-22 (2009).

[13] Brisbin S., Liu J., Boudreau J., Peng J., Evangelista M., & Chin-Sang I. A role for *C. elegans* Eph RTK signaling in PTEN regulation. *Dev Cell*. **17**, 459-69 (2009).

[14] Okuyama T., Inoue H., Ookuma S., Satoh T., Kano K., Honjoh S., Hisamoto N., Matsumoto K., & Nishida E. The ERK-MAPK pathway regulates longevity through SKN-1 and insulin-like signaling in *Caenorhabditis elegans*. *J Biol Chem*. **285**, 30274-81 (2010).

[15] Oh S.W., Mukhopadhyay A., Svrzikapa N., Jiang F., Davis R.J., & Tissenbaum H.A.. JNK regulates lifespan in *Caenorhabditis elegans* by modulating nuclear translocation of forkhead transcription factor/DAF-16. *Proc Natl Acad Sci U S A*. **102**, 4494-9 (2005).

[16] Curran S.P., Wu X., Riedel C.G., & Ruvkun G. A soma-to-germline transformation in long-lived *Caenorhabditis elegans* mutants. *Nature*. **459**, 1079-84 (2009).

[17] Smith-Vikos T., de Lencastre A., Inukai S., Shlomchik M., Holtrup B., & Slack F.J. MicroRNAs mediate dietary-restriction-induced longevity through PHA-4/FOXA and SKN-1/Nrf transcription factors. *Curr Biol*. **24**, 2238-2246 (2014).

[18] Murphy C.T., Lee S.J., & Kenyon C. Tissue entrainment by feedback regulation of insulin gene expression in the endoderm of *Caenorhabditis elegans*. *Proc Natl Acad Sci U S A*. **104**, 19046-19050 (2007).

[19] Hansen M., Hsu A.L., Dillin A., & Kenyon C.. New genes tied to endocrine, metabolic, and dietary regulation of lifespan from a *Caenorhabditis elegans* genomic RNAi screen. *PLoS Genet*. **1**, 119-28 (2005).

[20] Petrascheck M., Ye X., & Buck L.B. An antidepressant that extends lifespan in adult *Caenorhabditis elegans*. *Nature*. 450, 553-6 (2007).

[21] Curran S.P., & Ruvkun G. Lifespan regulation by evolutionarily conserved genes essential for viability. *PLoS Genet*. **3**, e56 (2007).

[22] Goudeau J., Bellemin S., Toselli-Mollereau E., Shamalnasab M., Chen Y., & Aguilaniu H. Fatty acid desaturation links germ cell loss to longevity through NHR-80/HNF4 in *C. elegans*. *PLoS Biol*. **9**, e1000599 (2011).

[23] McCormick M., Chen K., Ramaswamy P., & Kenyon C.. New genes that extend *Caenorhabditis elegans*' lifespan in response to reproductive signals. *Aging Cell*. **11**, 192-202 (2012).

[24] Yee C., Yang W., & Hekimi S. The intrinsic apoptosis pathway mediates the pro-longevity response to mitochondrial ROS in *C. elegans*. *Cell*. **157**, 897-909 (2014).
