# Supplementary material for: Phosphorothioate-DNA bacterial diet reduces the ROS levels in C. elegans while improving locomotion and longevity
Source: Commun Biol. 2021 Nov 25;4:1335. doi: 10.1038/s42003-021-02863-y (PMC8617147; doi:10.1038/s42003-021-02863-y)
Supplement: Supplementary file 2 — Description of Additional Supplementary Files [file 42003_2021_2863_MOESM2_ESM.pdf]

## Description of Additional Supplementary Files

**File name:** Supplementary Data 1

**Description:** Categories enriched of up-regulated genes at Day 4.

**File name:** Supplementary Data 2

**Description:** Categories enriched of up-regulated genes at Day 12.

**File name:** Supplementary Data 3

**Description:** Categories enriched of down-regulated genes at Day 4.

**File name:** Supplementary Data 4

**Description:** Categories enriched of down-regulated genes at Day 12.

**File name:** Supplementary Data 5

**Description:** KEGG pathways enriched of up-regulated genes at Day 4

**File name:** Supplementary Data 6

**Description:** KEGG pathways enriched of up-regulated genes at Day 12

**File name:** Supplementary Data 7

**Description:** KEGG pathways enriched of down-regulated genes at Day 4

**File name:** Supplementary Data 8

**Description:** Source data for Figures 1a-c, 2-3, and 4a.
